# Excess folate elevates formaldehyde genotoxicity in cell lines but not in mice or humans

**DOI:** 10.1038/s44321-026-00504-7

**Published:** 2026-08-12

**Authors:** Christopher Mellor, Brandon T James, Saeideh Azad, Emma P Kosmeder, Chloe T Purello, Monika Singh, Victoria Simon, Nicholas W Cheng, Jessica Niedermaier, Guillermo Burgos-Barragan, John O Blenis, Anthony R Mato, Martha S Field, Meng Wang

**Affiliations:** 1 Division of Nutritional Sciences, Cornell University, Ithaca, NY 14853 USA; 2 Cayuga Cancer Center, Cayuga Health, Ithaca, NY USA; 3 Sandra and Edward Meyer Cancer Center, Weill Cornell Medicine, New York, NY USA; 4 Department of Pharmacology, Weill Cornell Medicine, New York, NY USA; 5 Department of Biochemistry, Weill Cornell Medicine, New York, NY USA; 6 Department of Biomedical and Translational Sciences, College of Veterinary Medicine, Cornell University, Ithaca, NY 14853 USA

**Keywords:** Cancer, Haematology, Metabolism

## Abstract

Folate metabolites are chemically unstable: spontaneous decomposition releases formaldehyde, a genotoxin in blood stem cells and a human carcinogen. Despite this, folic acid consumption frequently exceeds the Recommended Dietary Allowance and is prescribed at high doses for patients with blood disorders. However, the impact of excess folate on endogenous formaldehyde genotoxicity in vivo has not been studied. We find that excess tetrahydrofolate (THF) treatment of cell lines elevates formaldehyde-DNA adducts and genotoxicity. To test this in vivo, we fed a high-folic acid diet (10-fold above standard) to mice with heightened sensitivity to formaldehyde: detoxification-impaired *Adh5*^*-/-*^ mice, and Fanconi anemia DNA repair mutants *Fanca*^*-/-*^ and *Fancj*^*-/-*^. In contrast to cell lines, elevated tissue THF was not associated with increased formaldehyde-DNA adducts nor blood stem cell attrition. Finally, in cancer patients, high-dose folic acid therapy elevated plasma folic acid but did not increase formaldehyde-DNA adducts in peripheral blood mononuclear cells. In conclusion, increased folate in vivo does not elevate endogenous formaldehyde genotoxicity in sensitized mouse models or humans.

## Synopsis

Folates can decompose to release formaldehyde, a genotoxin and a human carcinogen, raising concern over high-dose folic acid supplementation as a source of formaldehyde. Here, excess folic acid intake did not elevate formaldehyde-DNA damage in formaldehyde-sensitized mice or cancer patients.

- Reduced folates (THF, DHF), but not folic acid, were shown to elevate formaldehyde-DNA adducts by ~10-fold in cultured Adh5-/- cells, as measured by LC-MS.
- In Adh5-/- mice fed 20 mg/kg folic acid for 8 weeks, liver folates were elevated 1.5- to 2-fold, yet formaldehyde-DNA adducts and γH2AX were not increased.
- In formaldehyde-hypersensitive Fanconi anemia mutants (Fanca-/-, Fancj-/-), neither blood stem cell (HSPC) attrition nor micronucleated erythrocytes (MN-NCE) were increased by a high-folic acid diet (HFD).
- In cancer patients, PBMC formaldehyde-DNA adducts were not elevated by high-dose folic acid despite raised plasma folic acid, with no plasma folic acid-formaldehyde correlation detected.

## Abstract (web summary)

Folates can decompose to release formaldehyde, a genotoxin and a human carcinogen, raising concern over high-dose folic acid supplementation as a source of formaldehyde. Here, excess folic acid intake did not elevate formaldehyde-DNA damage in formaldehyde-sensitized mice or cancer patients.

## Graphical abstract

**Figure:**
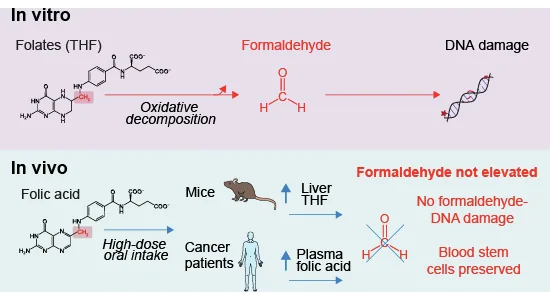

The paper explained **Problem**

Folic acid is consumed by millions and frequently prescribed at high doses to patients with blood disorders or cancer. However, excess folic acid supplementation has been associated with increased DNA damage, raising concerns over the safety of high-dose intake. One potential mechanism is that folate metabolites are chemically unstable and can decompose to release formaldehyde, a genotoxin and human carcinogen. Blood-forming (hematopoietic) stem cells are particularly vulnerable to formaldehyde, depending on formaldehyde-detoxifying enzymes and Fanconi anemia DNA repair to protect their genome. Whether excess folic acid increases formaldehyde-driven DNA damage in the body, especially in these vulnerable patients, had not been tested.

**Results**

In cultured cells, treatment with the reduced folate metabolite tetrahydrofolate increased formaldehyde-DNA damage, whereas folic acid did not. To test whether excess dietary folic acid causes this damage in vivo, mice were fed ten times the standard amount for 8 weeks, including strains engineered to be highly sensitive to formaldehyde through loss of detoxification enzymes or Fanconi anemia DNA repair. Despite increased tissue folate, formaldehyde-DNA adducts did not rise in any tissue, and blood stem cells were unharmed. In cancer patients taking high-dose folic acid, blood folic acid levels were elevated, but formaldehyde-DNA adducts in blood cells did not increase.

**Impact**

High-dose folic acid does not measurably increase formaldehyde-related DNA damage in vivo, even in highly sensitized settings. This provides evidence-based reassurance for the short-term high-dose folic acid given to patients, such as Fanconi anemia patients, during bone marrow transplantation or chemotherapy.

## Introduction

Folic acid is among the most widely consumed micronutrients worldwide. In the United States, an estimated 35% of adults take folic acid supplements (Bailey et al, 2010), and, combined with mandatory food fortification, roughly 7 million have intakes exceeding the tolerable upper intake level (Orozco et al, 2016). In clinical practice, folic acid is one of the most commonly prescribed drugs in hematology, on the rationale that rapidly dividing hematopoietic cells require additional one-carbon units for nucleotide biosynthesis. 1–5 mg folic acid daily (2.5–12.5 times the recommended dietary allowance) is standard-of-care for ~100,000 sickle cell disease patients in the US (Hassell, 2010), and routinely prescribed for patients with hemolytic anemias (Hill et al, 2017), hemoglobinopathies (UK Thalassaemia Society, 2023), bone marrow failure and for patients receiving chemotherapy or transplants. Notably, doses above 1 mg/day exceed the Tolerable Upper Intake Level (Subcommittee on Upper Reference Levels of Nutrients and Standing Committee on the Scientific Evaluation of Dietary Reference Intakes and its Panel on Folate and Other B Vitamins and Choline, 2000), yet the consequences of excess folic acid exposure remain understudied (Maruvada et al, 2020). Of concern, experimental evidence indicates that high-dose oral folate increases genomic instability in hematopoietic cells (García-Calderón et al, 2018; Henry et al, 2017) and mutagenesis in rodent models (Cao et al, 2023).

One mechanism that could explain folate-associated genotoxicity is the release of formaldehyde from folate metabolism (Burgos-Barragan et al, 2017; Mellor et al, 2025). Formaldehyde is a well-recognized human carcinogen and abundant endogenous genotoxin (Valverde-Santiago and Pontel, 2025; Wang et al, 2022). Hematopoietic stem and progenitor cells (HSPCs) are particularly sensitive, requiring formaldehyde detoxification enzymes ALDH2 and ADH5, and DNA repair to maintain genome stability (Wang et al, 2022). Mice lacking both detoxification enzymes or either enzyme combined with loss of Fanconi anemia DNA repair develop spontaneous bone marrow failure and leukemia from HSC attrition. In children, deficiencies in these pathways present with bone marrow failure and leukemia (Auerbach, 2009; Dingler et al, 2020; Oka et al, 2020).

There is a direct biochemical link between folates and formaldehyde. Following absorption, folic acid undergoes stepwise enzymatic reduction to dihydrofolate (DHF) and tetrahydrofolate (THF), which receives and shuttles one-carbon units through a series of cyclic enzymatic interconversions between 5,10-methylene-THF, 5,10-methenyl-THF and 10-formyl-THF to support thymidine and purine synthesis. In addition, the irreversible conversion of 5,10-methylene-THF to 5-methyl-THF provides the one-carbon unit for methionine synthesis, supporting formation of the universal methyl donor S-adenosylmethionine and, via transsulfuration of homocysteine, the redox regulator glutathione (Ducker and Rabinowitz, 2017; Fox and Stover, 2008). In vitro, certain folate metabolites—THF, DHF, and 5,10-methylene-THF—can release formaldehyde through oxidative decomposition (Burgos-Barragan et al, 2017; Chippel and Scrimgeour, 1970). Excess oral folic acid supplementation could therefore elevate these folate metabolites, thereby increasing formaldehyde-DNA damage in hematopoietic cells. This is particularly concerning for hematology patients whose HSCs are already compromised. For example, Fanconi anemia-deficient HSPCs are sensitive to formaldehyde-DNA damage (Pontel et al, 2015), yet patients are frequently administered high-dose oral folate to support hematopoiesis. Despite the scale of exposure, the effect of high-dose oral folic acid on endogenous formaldehyde genotoxicity has not been studied in vivo. To address this, we tested whether high-dose oral folic acid elevates formaldehyde-DNA adducts and compromises HSPCs using multiple mouse models with defective formaldehyde detoxification or FA DNA repair, and validated our findings in cancer patients receiving high-dose folic acid therapy.

## Results and discussion

**Figure EV1:**
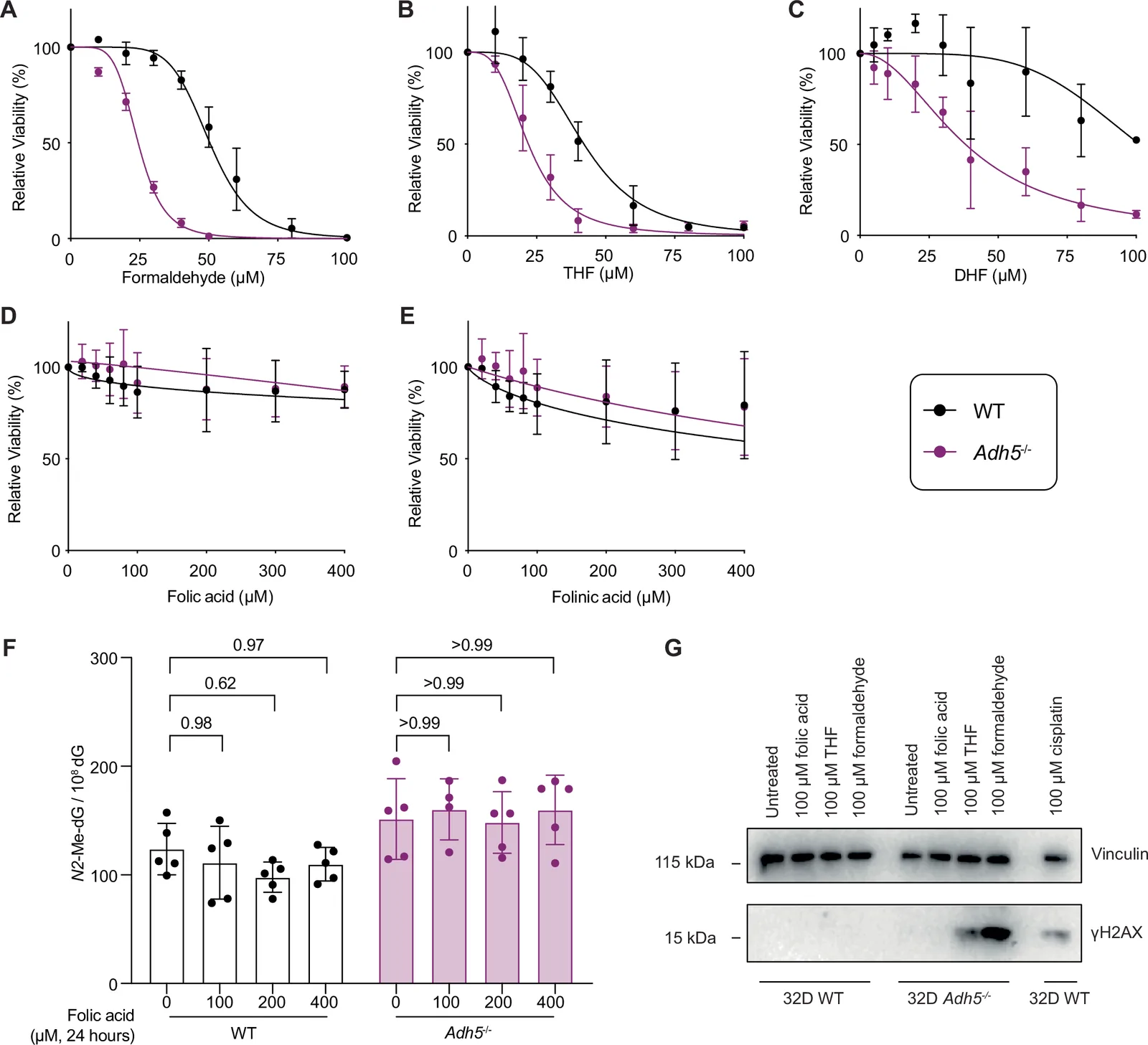
32D *Adh5*^*−/−*^ cells are sensitive to decomposition-prone folate derivatives, but not folic acid. (**A**–**E**) Cell survival following 72 h treatment of 32D WT and *Adh5*^*−/−*^ cells with formaldehyde (**A**), decomposition-prone folates (THF: **B**, DHF: **C**) and decomposition-resistant folates (folic acid: **D**, folinic acid: **E**). (**A**), *n* = 2; (**B**–**E**), *n* = 3. Mean ± SD. (**F**) Measurement of formaldehyde-induced *N2*-Me-dG adducts by LC-MS in WT and *Adh5*^*−/−*^ 32D cells, following 24-hour treatment with folic acid. *n* = 4–5, mean ± SD. One-way ANOVA with Šidák multiple comparisons test. (**G**) Western blot showing H2AX serine 139 phosphorylation (γH2AX) in 32D WT and *Adh5*^*−/−*^ cells following 6-h treatment with folic acid, THF or formaldehyde. 6-h cisplatin treatment was used as an additional positive control. Source data are available online for this figure.

**Figure 1:**
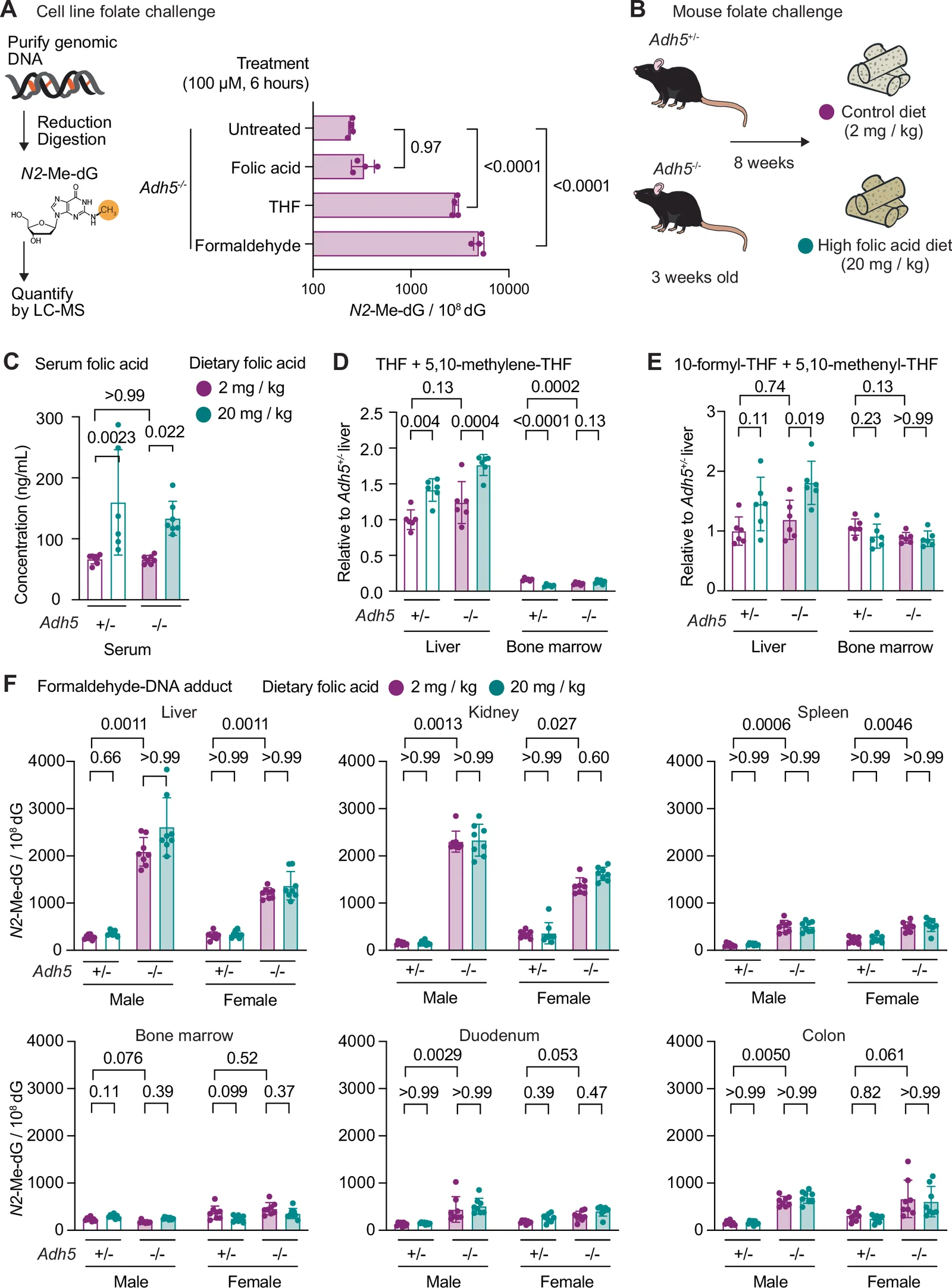
Excess folate elevates formaldehyde-induced DNA adducts in cell lines but not in mice. (**A**) Measurement of formaldehyde-induced *N2*-Me-dG adducts by LC-MS in *Adh5*^*−/−*^ 32D cells, following 6-h treatment with folic acid, THF or formaldehyde. *n* = 4, mean ± SD. One-way ANOVA with Šidák multiple comparisons test. (**B**) Schematic showing the challenge of C57BL/6 J *Adh5*^*+/−*^ and *Adh5*^*−/−*^ mice with a control diet (2 mg/kg) or high-folic acid diet (HFD) (20 mg/kg) for 8 weeks, commencing at weaning (3 weeks of age). (**C**) Quantification of serum folic acid. *n* = 7, 6, 7, 7 left-to-right, males, mean ± SD. One-way ANOVA with Šidák multiple comparisons test. (**D**,** E**) LC-MS quantification of folate derivatives in liver and bone marrow. Values were first normalized to the protein content of each tissue sample, then expressed relative to the corresponding folate derivative in *Adh5*^*+/−*^ liver. THF/5,10-methylene-THF and 10-formyl-THF/5,10-methenyl-THF pooled due to the spontaneous interconversion, which can occur during sample preparation. *n* = 6, males, mean ± SD. One-way ANOVA with Šidák multiple comparisons test. (**F**) Measurements of formaldehyde-induced *N2*-Me-dG adducts. *n* = 8 per sex, mean ± SD. Kruskal–Wallis test with Dunn multiple comparisons test. Source data are available online for this figure.

Natural folates in foods consist of reduced THF derivatives susceptible to spontaneous decomposition to release formaldehyde (Burgos-Barragan et al, 2017; Chippel and Scrimgeour, 1970). In contrast, synthetic folic acid is an oxidized precursor converted to THF by dihydrofolate reductase following absorption. We initially compared folic acid to THF for susceptibility to release formaldehyde through decomposition using *Adh5*-knockout 32D cells to leverage their increased sensitivity to formaldehyde. Consistent with prior studies (Burgos-Barragan et al, 2017; García-Calderón et al, 2018), *Adh5*^−/−^ 32D cells exhibited reduced growth and viability when treated with decomposition-prone folates (DHF, THF), but not with folic acid or a related synthetic folate, folinic acid (Fig. EV1A–E). To directly quantify cellular formaldehyde exposure from folates, we used ultrasensitive liquid chromatography-mass spectrometry (LC-MS) to measure formaldehyde-induced *N2-*hydroxymethyldeoxyguanosine (*N2*-Me-dG) DNA adducts, the current gold-standard assay to quantify endogenous formaldehyde exposure (Dingler et al, 2020; Pontel et al, 2015). THF induced a 10-fold elevation in *N2*-Me-dG adducts following 6-hour treatment, while folic acid did not elevate adducts at 6 or 24 h (Fig. 1A and EV1F). Consistent with previous observations (García-Calderón et al, 2018), THF treatment of *Adh5*^−/−^ 32D cells induced serine 139 phosphorylation of H2AX (γH2AX), a cellular marker of DNA damage and repair (Fig. EV1G). However, this was not observed following treatment with folic acid. A possible explanation for the absence of elevated endogenous formaldehyde-DNA adducts and γH2AX in 32D cells under supraphysiological folic acid supplementation is that folic acid uptake is saturated. Consistent with this, 32D cells express 10-fold lower levels of the high-affinity, folic acid–preferring folate receptors *Folr1* and *Folr2* than of *Slc19a1* (Appendix Fig. S1), which prefers reduced folates (Zhao et al, 2009). Consequently, to overcome this limitation of the cell culture model, we proceeded to model excess oral folic acid intake in mice to test the contribution of physiological gastrointestinal absorption of folic acid and its conversion to decomposition-prone reduced folates as a source of endogenous formaldehyde.

**Figure EV2:**
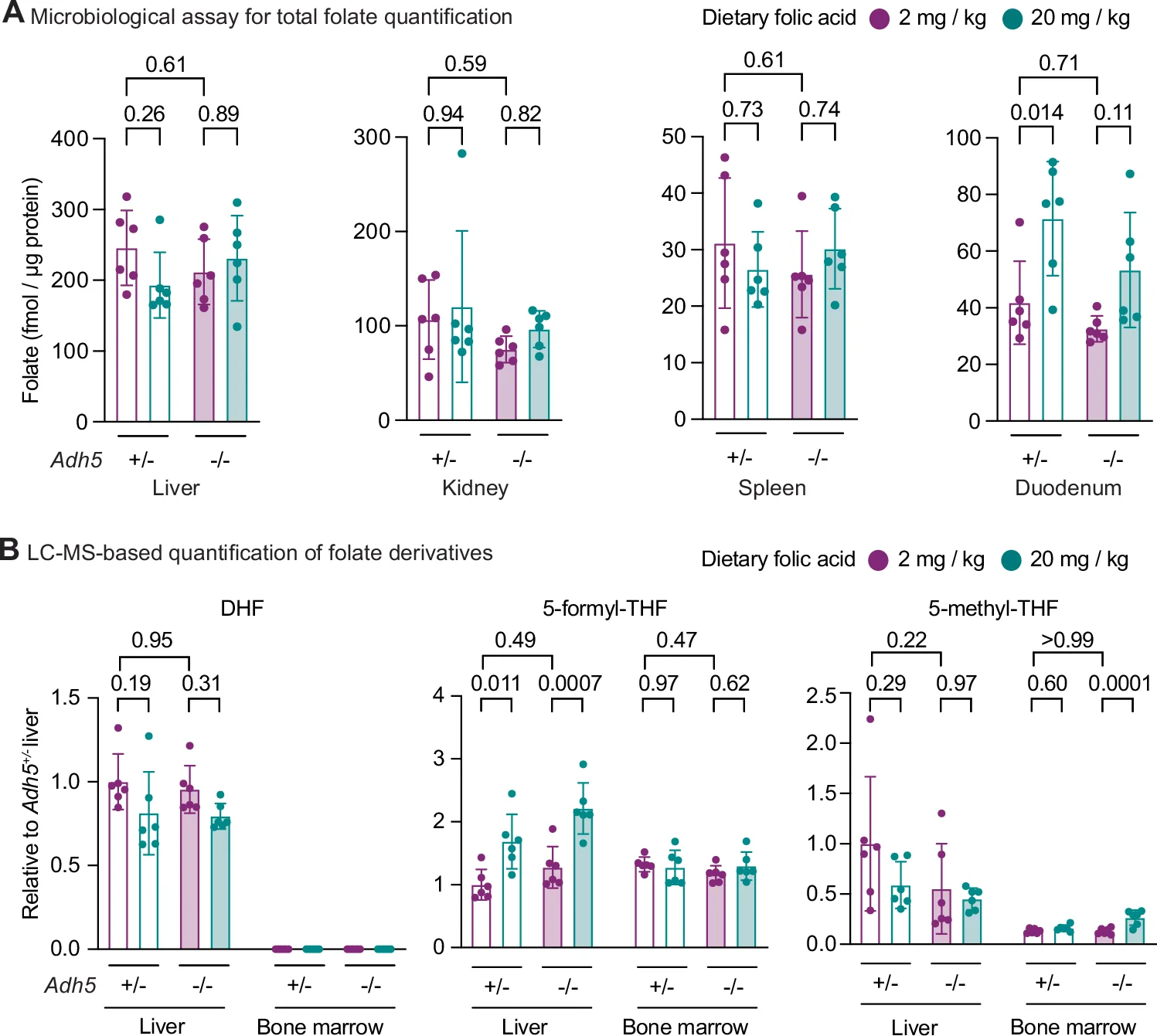
Quantification of tissue folate content in *Adh5*^*+/−*^ and *Adh5*^*−/−*^ mice fed a high-folic acid diet. (**A**) Total folate concentrations in the liver, kidney, spleen and duodenum of *Adh5*^*+/−*^ and *Adh5*^*−/−*^ mice fed a control diet or HFD for 8 weeks, quantified using the *Lactobacillus casei* microbiological assay. *n* = 6, male, mean ± SD. One-way ANOVA with Šidák multiple comparisons test. (**B**) LC-MS quantification of folate derivatives in liver and bone marrow. Values were first normalized to the protein content of each tissue sample, then expressed relative to the corresponding folate derivative in *Adh5*^*+/−*^ liver. *n* = 6, male, mean ± SD. One-way ANOVA with Šidák multiple comparisons test. Source data are available online for this figure.

**Figure EV3:**
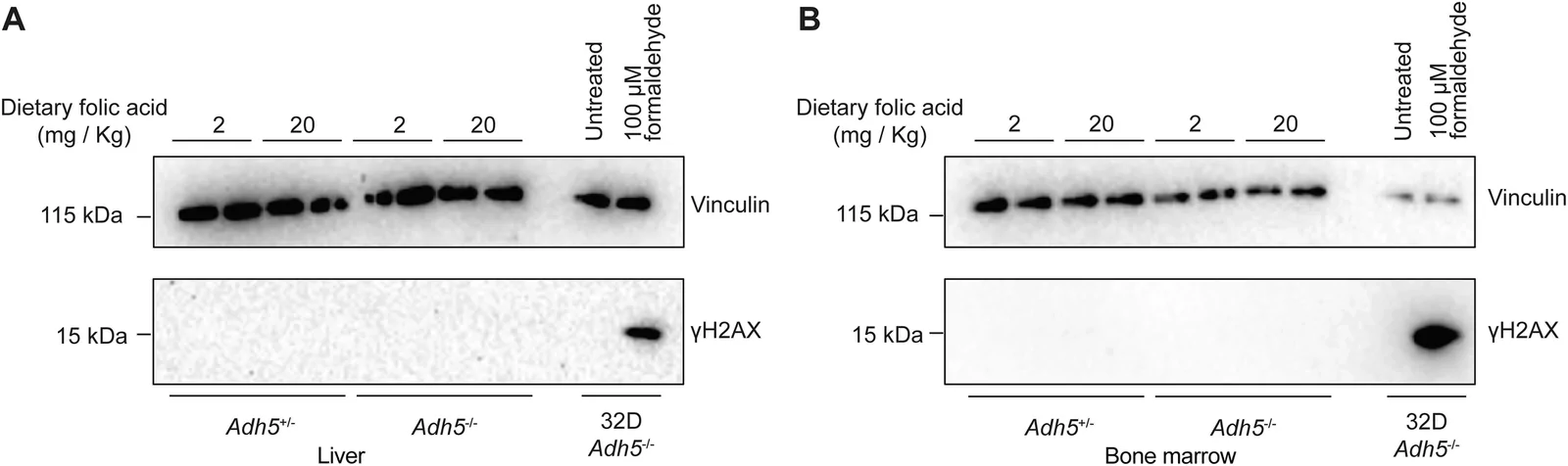
No detectable γH2AX signal in tissues of *Adh5*^*−/−*^ mice fed a high-folic acid diet. Western blots showing no detectable H2AX serine 139 phosphorylation (γH2AX) in the liver (**A**) and bone marrow (**B**) of 11-week-old male *Adh5*^*+/−*^ and *Adh5*^*−/−*^ mice fed a control diet or HFD for 8 weeks. 32D *Adh5*^*−/−*^ cells treated with 100 µM formaldehyde for 6 h were used as a positive control. Source data are available online for this figure.

We fed mice with intact (*Adh5*^+/−^) and compromised (*Adh5*^−/−^) formaldehyde detoxification either a control diet (2 mg/kg folic acid, standard in AIN-93 diets (Reeves et al, 1993)) or a high-folic acid diet (HFD, 20 mg/kg folic acid) for 8 weeks (Fig. 1B), which has previously been shown to elevate folate metabolites in vivo (Heyden et al, 2024; Munezero et al, 2025). HFD-fed mice exhibited increased serum folic acid (Fig. 1C). Although total organ folate was not significantly different by microbiological assay except in the small intestine (Fig. EV2A), LC-MS revealed significant alterations in the folate compositions in the liver, where we observed 1.5- to 2-fold increased concentration of the decomposition-prone folates (Figs. 1D,E and EV2B). Bone marrow showed lower folates than liver, with no increase in the concentration of decomposition-prone folates. We next tested if the elevation in decomposition-prone folates from the liver of HFD-fed mice was associated with increased formaldehyde-DNA adducts. While *N2*-Me-dG adducts were elevated by ADH5 deficiency as previously observed (Dingler et al, 2020; Pontel et al, 2015), strikingly, HFD did not increase formaldehyde-DNA adducts in the liver from *Adh5*^+/−^ or *Adh5*^−/−^ mice. Adduct levels also remained unchanged in spleen, bone marrow, kidney, duodenum and colon (Fig. 1F). Consistent with the lack of elevated adducts, we also observed no induction of γH2AX by immunoblotting in either the liver or bone marrow of *Adh5*^+/−^ or *Adh5*^−/−^ mice fed HFD (Fig. EV3). Therefore, HFD increases decomposition-prone folates without increasing endogenous formaldehyde in mice.

**Figure 2:**
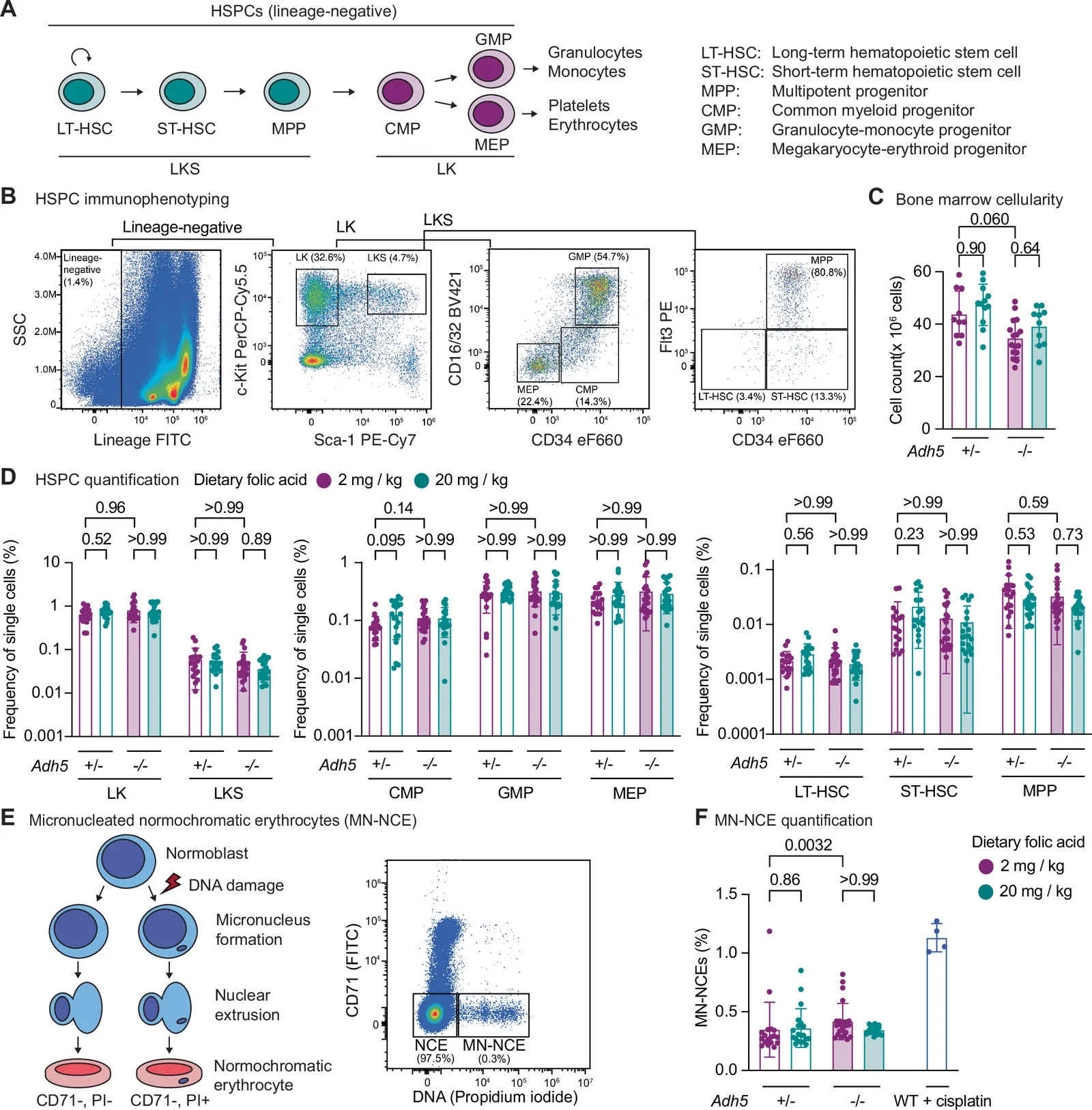
High folic acid diet does not cause HSPC attrition. (**A**) A map of the HSPC differentiation hierarchy (populations not presented in this study excluded). LKS: lineage-negative, c-Kit-positive, Sca-1-positive; LK: lineage-negative, c-Kit-positive. (**B**) A gating strategy to quantify HSPC abundance in mouse bone marrow by flow cytometry. (**C**) Bone marrow cellularity in *Adh5*^*+/−*^ and *Adh5*^*−/−*^ mice challenged with HFD. *n* = 10, 12, 16, 10 left-to-right. (**D**) Quantification of HSPC populations, assessed using the aforementioned gating strategy, in *Adh5*^*+/−*^ and *Adh5*^*−/−*^ mice challenged with HFD. *n* = 18, 20, 23, 21 left-to-right. (**E**) Schematic showing the process by which micronucleated normochromatic erythrocytes (MN-NCE) are generated during erythropoiesis, and how these can be quantified by flow cytometry. (**F**) The abundance of MN-NCE, as a percentage of total NCE (MN-NCE + NCE), in *Adh5*^*+/−*^ and *Adh5*^*−/−*^ mice challenged with HFD. *n* = 10, 12, 16, 10 left-to-right. WT mice treated with cisplatin (5 mg/kg), 48 h before collection of blood, used as a positive control (*n* = 4). All plots represent merged data from both sexes, mean ± SD. Kruskal–Wallis test with Dunn multiple comparisons test. Source data are available online for this figure.

We next tested the impact of HFD on HSPCs (Fig. 2A,B), which suffer severe attrition under elevated formaldehyde genotoxicity (Dingler et al, 2020; Pontel et al, 2015; Wang et al, 2023). We found *Adh5*^+/−^ or *Adh5*^−/−^ mice fed HFD showed no changes in bone marrow cellularity (Fig. 2C) or HSPC frequencies, including long-term and short-term HSCs, multipotent progenitors and myeloid progenitors (Fig. 2D). Consistent with intact HSPCs, HFD had no impact on peripheral blood counts (Appendix Fig. S2). We also measured the levels of micronuclei-containing normochromatic erythrocytes (MN-NCEs) (Fig. 2E), a marker of HSPC genomic instability associated with formaldehyde genotoxicity (Dingler et al, 2020). While *Adh5*^−/−^ mice showed increased micronuclei compared to *Adh5*^+/−^, HFD had no impact in either genotype (Fig. 2F).

**Figure 3:**
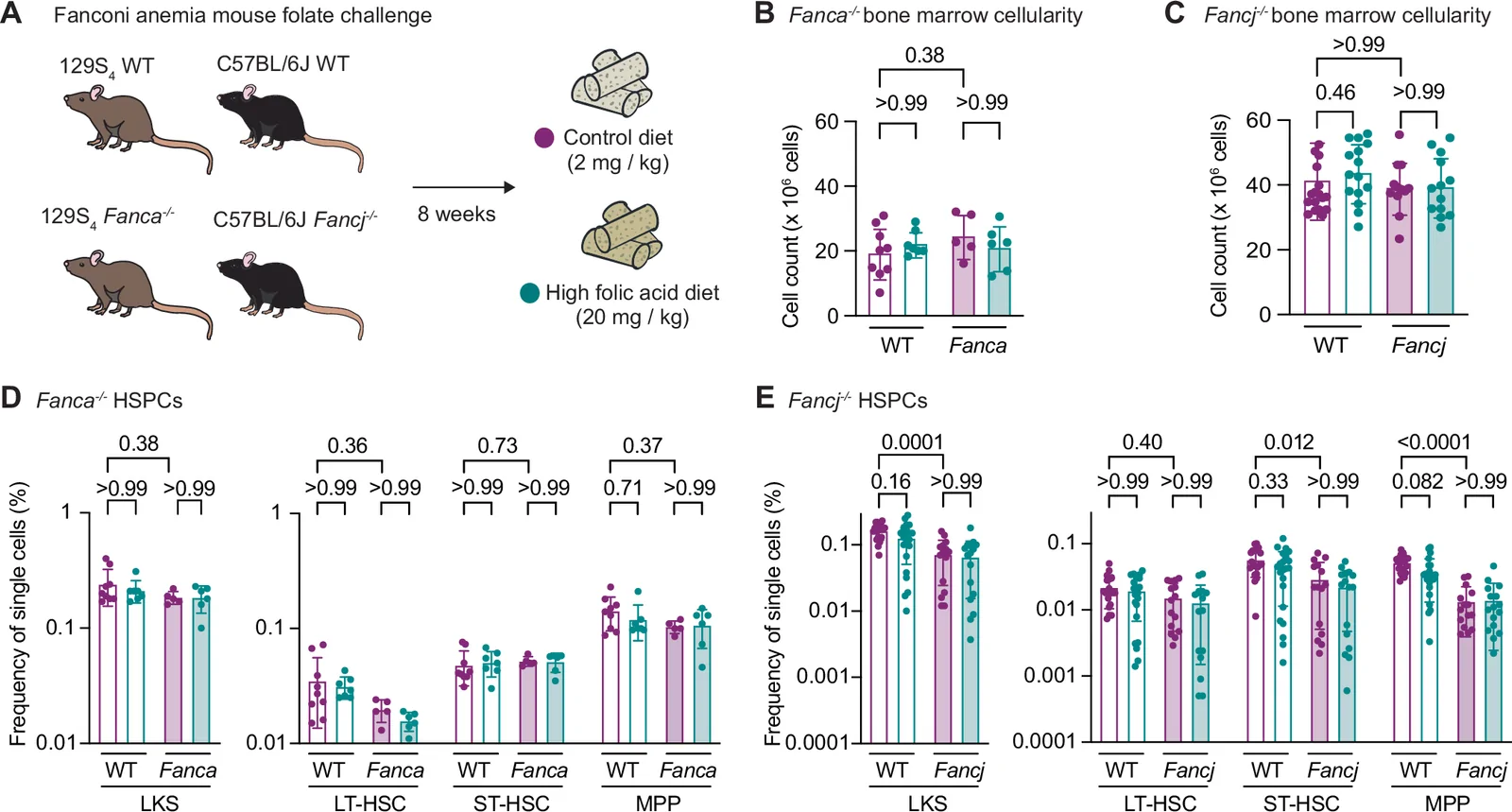
High folic acid diet does not cause HSPC attrition in Fanconi anemia mouse models. (**A**) Schematic showing the challenge of 129/S_4_ *Fanca*^*−/−*^ (5- to 19-week-old) and C57BL/6 J *Fancj*^*−/−*^ (3-week-old) mice with a HFD for 8 weeks. (**B**) Bone marrow cellularity in *Fanca*^*−/–*^ mice. *n* = 9, 7, 5, 6 left-to-right. (**C**) Bone marrow cellularity in *Fancj*^*−/−*^. *n* = 19, 15, 12, 13 left-to-right. (**D**) Quantification of HSPC populations in *Fanca*^*−/−*^ mice. *n* = 9, 7, 5, 6 left-to-right. (**E**) Quantification of HSPC populations in *Fancj*^*−/−*^ mice. *n* = 20, 22, 15, 16 left-to-right. All plots represent merged data from both sexes, mean ± SD. Kruskal–Wallis test with Dunn multiple comparisons test. Source data are available online for this figure.

**Figure EV4:**
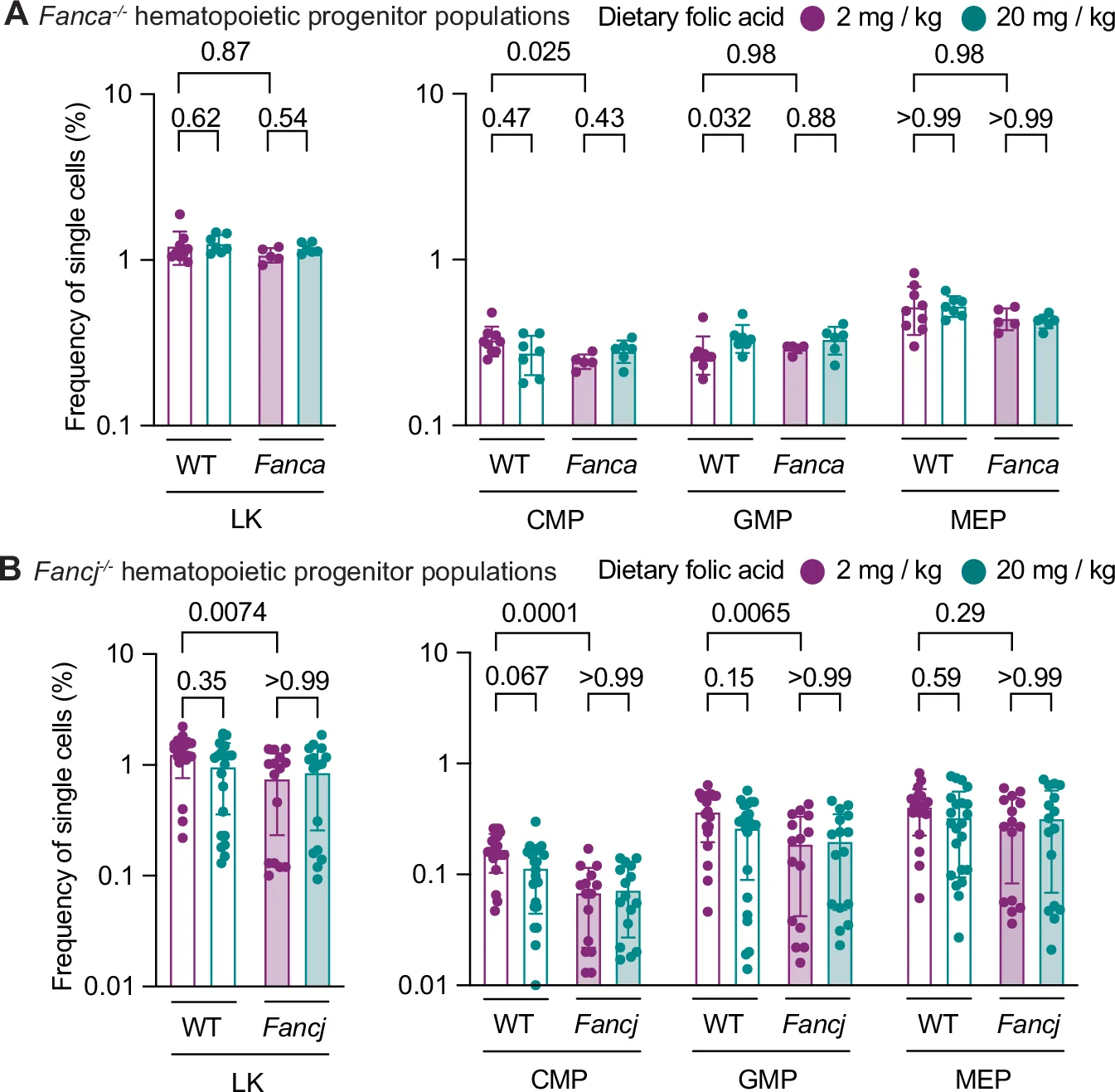
No change in hematopoietic progenitor frequencies in Fanconi anemia mouse models fed a high-folic acid diet. Bone marrow progenitor frequencies in 129/S_4_ *Fanca*^*−/−*^ and C57BL/6 J *Fancj*^*−/−*^ mice fed a HFD for 8 weeks, assessed by flow cytometry. (**A**) Quantification of hematopoietic progenitor populations in *Fanca*^*−/−*^ mice. 5= 9, 7, 5, 6 left-to-right. (**B**) Quantification of hematopoietic progenitor populations in *Fancj*^*−/−*^ mice. *n* = 20, 22, 15, 16 left-to-right. All plots represent merged data from both sexes, mean ± SD. Kruskal–Wallis test with Dunn multiple comparisons test. Source data are available online for this figure.

**Figure EV5:**
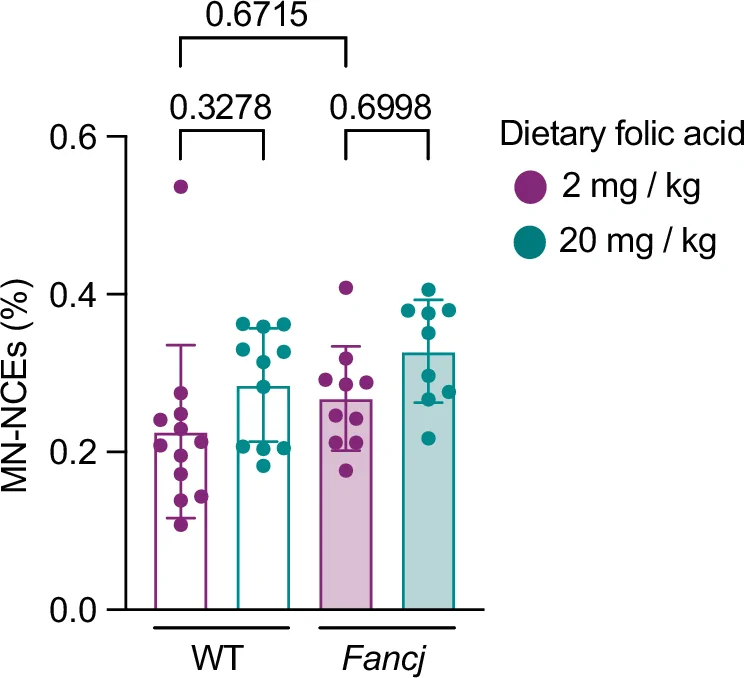
No change in micronucleated normochromatic erythrocytes in *Fancj*^*−/−*^ mice fed a high-folic acid diet. The abundance of MN-NCE, as a percentage of total NCE (MN-NCE + NCE), in C57BL/6 J *Fancj*^*−/−*^ mice challenged with HFD for 8 weeks. *n* = 12, 11, 10, 9 left-to-right, merged data from both sexes, mean ± SD. Kruskal–Wallis test with Dunn multiple comparisons test. Source data are available online for this figure.

In parallel to formaldehyde detoxification, HSPCs also rely on Fanconi anemia DNA repair to protect their genome against formaldehyde-induced DNA damage (Wang et al, 2022) (Fig. 3A). To rigorously exclude high-dose oral folic acid as a source of formaldehyde genotoxicity in HSPCs, we fed HFD to two Fanconi anemia mouse models representing distinct steps of the pathway: FANCA (core complex) and FANCJ (downstream lesion processing). While the Fanconi anemia-null mice exhibit reduced HSPC frequencies compared to wildtype mice, HFD had no detectable impact on bone marrow cellularity (Fig. 3B,C), HSPC frequencies (Figs. 3D, E and EV4), peripheral blood counts (Appendix Fig. S3) or MN-NCEs (Fig. EV5). Altogether, oral high-dose folate does not cause HSPC attrition in formaldehyde detoxification-deficient or FA-deficient mice.

**Figure 4:**
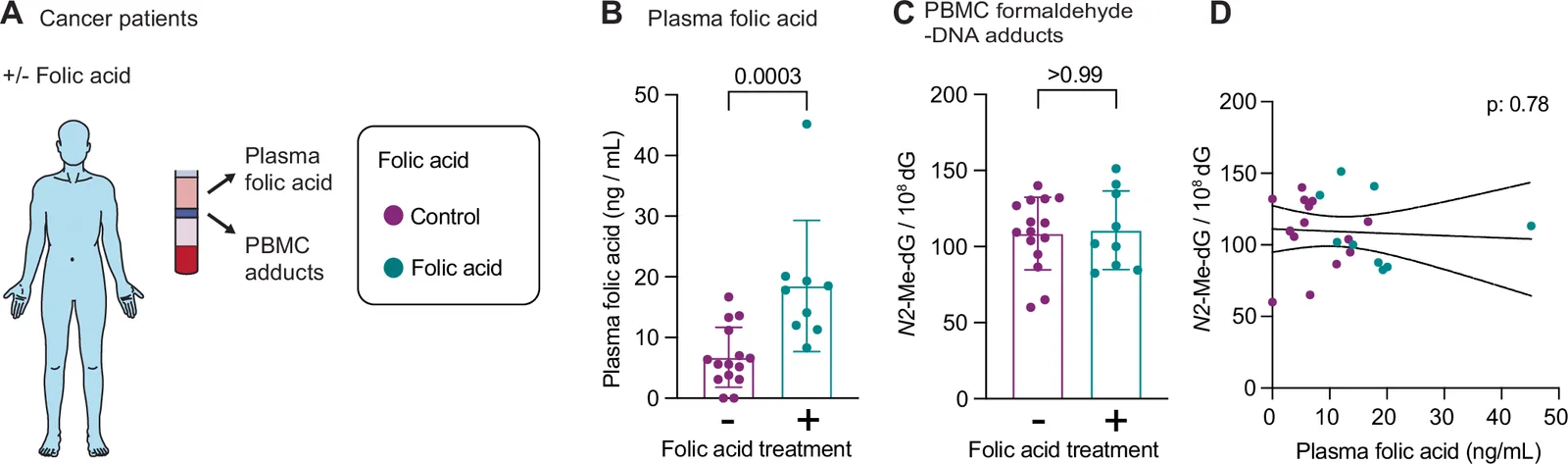
High-dose folic acid therapy in cancer patients does not elevate formaldehyde-DNA adducts in PBMCs. (**A**) Schematic showing the collection and analyses of blood samples from patients treated with high-dose folic acid (0.4–2 mg daily, ≥2 weeks), or a control group not taking folic acid therapy, as per standard-of-care cancer treatment. Demographic data provided in Appendix Table S1. (**B**) Plasma folic acid levels in the control and high-dosage folic acid patient groups. *n* = 15, 9 left-to-right, mean ± SD. Mann–Whitney test. (**C**) Measurement of formaldehyde-induced *N2*-Me-dG adducts by LC-MS in PBMCs. *n* = 15, 9 left-to-right, mean ± SD. Mann–Whitney test. (**D**) Correlation between plasma folic acid levels and PBMC *N2*-Me-dG adducts in patient samples. Control: purple (*n* = 15), folic acid supplemented: green (*n* = 9). Simple linear regression, *p* value indicates the probability that the slope is significantly non-zero. Curved lines indicate 95% confidence intervals. Source data are available online for this figure.

Finally, to validate our findings clinically, we compared formaldehyde-DNA adducts in peripheral blood mononuclear cells (PBMCs) from cancer patients taking high-dose folic acid (0.4–2 mg daily for ≥2 weeks) to cancer patients not on folic acid therapy (Fig. 4A; Appendix Table S1). We recruited 24 patients (9 taking folic acid, 15 controls) receiving standard-of-care treatment at the Cayuga Cancer Center. High-dose folic acid increased plasma folic acid levels (Fig. 4B) but did not increase formaldehyde-DNA adduct levels in PBMCs (Fig. 4C). Furthermore, we find no correlation between individual plasma folic acid and PBMC adducts (Fig. 4D). In summary, high-dose folic acid treatment in humans does not increase endogenous formaldehyde in hematopoietic cells.

Folate metabolites can generate formaldehyde through spontaneous chemical decomposition, but the effect of elevated folate metabolites on endogenous formaldehyde genotoxicity has not been studied in vivo. To address this, we used ultrasensitive LC-MS quantification of formaldehyde-DNA adducts to find that while direct THF treatment of cell lines elevated formaldehyde genotoxicity, increased THF accumulation in vivo from oral folic acid supplementation did not elevate endogenous formaldehyde in mouse models or humans. Our findings are consistent across multiple model systems deficient in formaldehyde detoxification or DNA repair, which amplify sensitivity to both endogenous formaldehyde elevation and its genotoxic impact on HSPCs. Together with the human data, these results provide evidence-based reassurance that high-dose folate therapy does not exacerbate formaldehyde-mediated genotoxicity. This has direct relevance for Fanconi anemia patients requiring short-term high-dose folic acid as supportive treatment during bone marrow transplantation or chemotherapy.

There are limitations to our study. First, we only focus on formaldehyde-induced genotoxicity with a single timepoint of analysis after 8 weeks of HFD in mice. We therefore cannot exclude that a longer duration on HFD could precipitate formaldehyde genotoxicity from chronic accumulation of folates, which could have additive effects with other mechanisms of genotoxicity associated with excess oral folic acid, including oxidative damage, nucleotide imbalance and uracil misincorporation (Alnabbat et al, 2022; Henry et al, 2017; Heyden et al, 2024; Koseki et al, 2020). A previous study (Cao et al, 2023) used a longer HFD challenge (16 weeks) in mice to observe a 1.8-fold elevation in de novo mutation rates in their offspring. Despite this increased mutagenesis, there was no enrichment for any specific mutation patterns that could be attributed to formaldehyde (Blouin et al, 2025; Dingler et al, 2020; Kucab et al, 2019; Thapa et al, 2022). Second, we did not quantify formaldehyde-adducts in the oral cavity, a frequent site of cancer in Fanconi anemia patients (Altintas et al, 2023; Ramírez et al, 2025). It will be relevant to assess the genotoxic impact of formaldehyde arising from the decomposition of folates in food and/or microbiome through direct mucosal contact in the oral cavity. Third, the experimental systems used here do not model pregnancy, so caution is warranted in translating our findings to folic acid supplementation for pregnant people.

In contrast to the susceptibility of THF derivatives to release formaldehyde under in vitro conditions, it remains unclear why elevated THF derivatives from folic acid supplementation do not increase endogenous formaldehyde in vivo. One explanation is that cellular mechanisms prevent folate decomposition in vivo. Quinoid dihydropteridine reductase enzyme can repair partially oxidized folates before full decomposition can occur (Zheng et al, 2018). Furthermore, intracellular folates are predominantly sequestered on high-affinity binding proteins (Stover and Field, 2011), which could prevent the spontaneous decomposition that occurs readily in vitro. Together, these mechanisms may be sufficient to prevent formaldehyde release from folate decomposition. In conclusion, we show that oral folic acid supplementation does not measurably contribute to endogenous formaldehyde burden in vivo.

## Methods

**Table:** Reagents and tools table

Reagent/resource	Reference or source	Identifier or catalog number
**Experimental models**		
*Lacticaseibacillus rhamnosus*	ATCC	7469
32D WT cells	Wit et al, 2023	N/A
32D *Adh5* cells	Wit et al, 2023	N/A
C57BL/6 J *Adh5*^*tm1Stam*^	Liu et al, 2004	MGI ID: 3033711
C57BL/6J *Fancj*	Horan et al, 2024	N/A
129S_4_ *Fanca*	Noll et al, 2002	N/A
**Recombinant DNA**		
N/A		
**Antibodies**		
Vinculin polyclonal antibody	Proteintech	26520-1-AP
Phospho-Histone H2A.X (Ser139) Antibody	Cell Signaling Technology	2577
HRP-conjugated Goat Anti-Rabbit IgG (H + L)	Proteintech	SA00001-2
FITC anti-mouse CD3ε (cl 145-2C11)	BioLegend	100306
FITC anti-mouse CD4 (cl H129.19)	BioLegend	130308
FITC anti-mouse CD8a (cl 53-6.7)	BioLegend	100706
FITC anti-mouse/human CD11b (cl M1/70)	BioLegend	101206
FITC anti-mouse CD11c (cl N418)	BioLegend	117306
FITC anti-mouse/human CD45R/B220 (cl RA3-6B2)	BioLegend	103206
FITC anti-mouse FcεRIα (cl MAR-1)	BioLegend	134306
FITC anti-mouse Ly-6G/Ly-6C (Gr-1) (cl RB6-8C5)	BioLegend	108406
FITC anti-mouse TER-119 /Erythroid cells (cl TER-119)	BioLegend	116206
PerCP/Cyanine5.5 anti-mouse CD117 (c-kit) (cl 2B8)	BioLegend	105824
PE/Cyanine7 anti-mouse Ly-6A/E (Sca-1) (cl D7)	BioLegend	108113
PE anti-mouse CD135 (cl A2F10)	BioLegend	135306
Anti-Mo CD34, eFluor 660 (cl RAM34)	Invitrogen	50-0341-82
Brilliant Violet 421 anti-mouse CD16/32 (cl 93)	BioLegend	101332
Brilliant Violet 605 anti-mouse CD127 (IL7-Rα) (cl A7R34)	BioLegend	135025
FITC anti-mouse CD71 (cl R17217)	BioLegend	113805
**Oligonucleotides and other sequence-based reagents**		
N/A		
**Chemicals, enzymes and other reagents**		
PBS Buffer Powder	VWR	76371-734
RPMI 1640 Medium, GlutaMAX Supplement	Gibco	61870036
Fetal bovine serum value FBS	Gibco	A525670
Penicillin-Streptomycin 10,000 U/mL	Gibco	15140122
Recombinant Murine interleukin-3 (IL-3)	PeproTech	213-13
16% formaldehyde solution	Thermo Scientific	28906
Folic acid	Thermo Scientific	216630100
DL-folinic acid	Cayman Chemical Company	20383
Dihydrofolic acid	Cayman Chemical Company	32750
Tetrahydrofolic acid	MedChemExpress	HY-14520
CellTiter 96 AQueous One Solution Cell Proliferation Assay	Promega	G3581
4x Laemmli Sample Buffer	Bio-Rad	1610747
2-mercaptoethanol	Sigma	M6250
RIPA Buffer	Proteintech	PR20135
Benzonase Nuclease	Millipore	E1014
Protease/Phosphatase Inhibitor Cocktail (100X)	Cell Signaling Technology	5872
Pierce BCA Protein Assay Kit	Themo Scientific	23227
SurePAGE Bis-Tris, 10×8, 4–12%, 15 wells	Genscript	M00654
PageRuler Prestained Protein Ladder	Thermo Scientific	26616
MOPS-SDS running buffer (20X)	National Diagnostics	EC-867
0.45 µm PVDF membrane	GVS	1212781
10x Tris/Glycine Buffer	Bio-Rad	1610771
TBS Buffer Powder	Accuris	EB1202
Tween 20	Research Products International	P20370
Carnation Instant Nonfat Dry Milk	Nestlé	N/A
Albumin, Bovine (BSA)	VWR	0332
Radiance Plus HRP Substrate	Azure	S1006
IMMULITE 2000 Folic Acid assay	Siemens	10380911
Folic acid Casei Medium	HIMEDIA	M543
HEPES	VWR	BP310
CHES	Alfa Aesar	A18047
Rat serum	VWR	103219-662
Sodium ascorbate	VWR	A7631
Gamma-glutamyl hydrolase Recombinant Protein	Novus Biologicals	NBP1-78898
Bond Elut-PH columns	Agilent	12102062
DC Protein Assay	Bio-Rad	5000111
5-methyl-THF ^13^C_5_	Synthesized in-house	N/A
Puregene Cell Lysis Solution	Qiagen	158116
Puregene Protein Precipitation Solution	Qiagen	158126
Proteinase K	Qiagen	RP107B-5
RNase A Solution	Qiagen	158924
Sodium cyanoborohydride	Oakwood Chemical	044871
Sodium acetate solution 3 M	Sigma	S7899
^15^N_5_-deoxyadenosine	Cambridge Isotope Technologies	N/A
^15^N_5_-*N2*-methyldeoxyguanosine	Kind gift of Ketan J Patel (University of Oxford)	N/A
Shrimp Alkaline Phosphatase (rSAP)	NEB	M0371
DNase I recombinant, RNase-free	Roche	04716728001
Phosphodiesterase I from *Crotalus adamanteus* venom	Sigma	P3243
3 K MWCO Micro Spin Filters	Analytical	8690-03
RBC Lysis Buffer (10X)	Cell Signaling Technology	46232S
Bond Elut-PH columns	Agilent	12102062
**Software**		
FlowJo v10	Water Bioscience	
XCalibur 4.1	Thermo Scientific	
SciexOS 3.4.5.828	Sciex	
**Other**		
EPOCH microplate reader	BioTek	
Chemidoc MP	Bio-Rad	
DxH 520 Hematology Analyzer	Beckman Coulter	
ViCell XR Cell Viability Analyser	Beckman Coulter	
Tissue Lyzer II	Qiagen	
Aurora CS	Cytek	
Attune NxT	Thermo Fisher	
IMMULITE 2000 XPi Immunoassay System	Siemens	
Vanquish UHPLC	Thermo Fisher	
Cadenza CD-C18 Length: 150 mm, ID: 2.0 mm, 3.0um	Imtakt	CD025
Q Exactive Orbitrap mass spectrometer	Thermo Fisher	
M5 microLC trap-elute system	Sciex	
Luna C18(2) trap column (5 µm particle size, 100 Å, 20 mm×0.30 mm)	Phenomenex	05M-4252-AC
Kinetex XB-C18 analytical column (2.6 µm particle size, 100 Å, 50 mm×0.3 mm)	Phenomenex	00B-4496-AC
Triple Quad™ 7500 LC-MS/MS System–QTRAP® Ready	Sciex	

### Cell culture

32D cells (a murine bone marrow progenitor cell line) (Greenberger et al, 1983; Valtieri et al, 1987) were grown in RPMI 1640 media supplemented with Glutamax, 10% Gibco Value Fetal Bovine Serum, 5 ng/mL murine IL-3 (Peprotech), and penicillin-streptomycin (Gibco). 32D *Adh5* knockout cells were generated as previously described (Wit et al, 2023). Reagents used for cell treatment were 16% formaldehyde solution (Thermo Scientific), folic acid (Thermo Scientific), DL-folinic acid (Cayman Chemical Company), tetrahydrofolic acid (MedChemExpress) and dihydrofolic acid (Cayman Chemical Company). Cell survival assays were performed by seeding cells at 0.05 × 10^6^ cells per mL in 96-well plates with the indicated concentration of drugs, assaying cell growth after 72 h using CellTiter 96 AQueous One Solution Cell Proliferation Assay (Promega).

### Mouse strains and dietary experiments

The *Adh5* knockout allele (Liu et al, 2004) (*Adh5*^*tm1Stam*^; MGI ID: 3033711, gifted by Dr. Jonathan Stamler) and the *Fancj* knockout allele (Horan et al, 2024) (*Fancj*^*-*^; kindly gifted by Dr. Paula Cohen) were maintained in a C57BL6/J background. The *Fanca* knockout allele (Noll et al, 2002) (kindly gifted by Dr. Markus Grompe) was maintained in a 129S_4_ background. In Fanconi anemia mouse models, mice homozygous for the WT allele (+/+) and heterozygotes (+/−) were pooled and classed as WT. Unless otherwise specified, all dietary challenge experiments used both male and female mice that were provided AIN-93G diets containing 2 or 20 mg folic acid/kg (Dyets) as their exclusive source of food from wean (3-week-old) for 8 weeks prior to euthanasia and collection of blood and tissues for analysis (11-week-old). Mice were housed in the AAALAC-certified Weill Hall barrier facility for accepted pathogens, maintained on a 10:14 h light-dark cycle in cages with constant air circulation, with food (ad libitum) and water. All mice were maintained in micro-isolator cages under virus/pathogen-free conditions. Cages contained Bed-o’-cobs bedding (Scotts 330BB), Bio-Serv standard hut (Scotts K3352), and Crinkle Nest Kraft (Scotts CNK), and a 1” × 1” nestlet (Scotts NES7200) for enrichment. Animal handling and procedures were performed following ethical approval by the Cornell Institutional Animal Care and Use Committee under protocol 2023–0054.

### Collection and processing of patient blood samples

Cancer patients were recruited at the Cayuga Cancer Center, Ithaca, NY, under an IRB-approved prospective biospecimen collection protocol (protocol CMC-001-01; PI: Dr. Anthony Mato). The study was conducted in accordance with the WMA Declaration of Helsinki and the Department of Health and Human Services Belmont Report, and approved by the respective IRBs at Cayuga Medical Center (IRB study number 0125AM) and Cornell University (IRB study number IRB0149864). All participants provided written informed consent prior to specimen collection. Eligible participants were adults (≥18 years) with a current or known history of cancer receiving standard-of-care treatment at the center. Patients were enrolled into two groups: those receiving high-dose folic acid supplementation (0.4–2 mg daily for ≥2 weeks) as part of their standard clinical care, and a control group of cancer patients not receiving folic acid therapy. Exclusion criteria included current inpatient status, active intensive chemotherapy regimens, active infections, and ECOG performance status >3. Enrolled patients were in remission or receiving maintenance therapy. De-identified demographic and clinical data, including age, sex, cancer diagnosis, and folic acid dose, were recorded (Appendix Table S1). A single peripheral blood sample (~6 mL) was collected in EDTA-anticoagulation tubes at the time of routine phlebotomy. De-identified specimens and clinical data were transferred to Cornell University for analysis. No investigators at Cornell University had direct contact with human subjects or access to identifiable patient information. Fresh whole blood (~6 mL per tube) collected in an EDTA-anticoagulation tube was diluted 1:1 with room temperature PBS. The mixture was homogenized by inverting the tube several times. Peripheral blood mononuclear cells (PBMCs) were isolated by density gradient centrifugation using Ficoll-Paque PLUS (Cytiva; density 1.077 g/mL). Ficoll (3 parts) was added to a 50 mL conical tube, and diluted blood (4 parts) was layered on top. Samples were centrifuged at 400 × *g* for 30 min at room temperature. The plasma layer was collected and stored at –80 °C for subsequent measurement of plasma folic acid, as described below. The PBMC interface was aspirated and transferred to a new sterile tube. Cells were washed twice by resuspension in 15 mL PBS followed by centrifugation at 400 × *g* for 10 min at room temperature. After the final wash, the supernatant was discarded, and the pellet was resuspended in 300 µL PBS. Total cell counts were determined using a Vi-CELL XR cell viability analyzer (Beckman Coulter). Cells were aliquoted into 2 mL microcentrifuge tubes at 5–7 × 10⁶ cells per tube, pelleting at 500 × *g* for 5 min at room temperature, and stored at –80 °C for subsequent DNA adduct analysis.

### Analysis of the hematopoietic system

Complete blood counts (CBCs) were taken using a Beckman Coulter DxH 520, using blood obtained from terminal cardiac puncture, collected in an EDTA-containing microtube. Bone marrow progenitors were analyzed as previously described (Dingler et al, 2020).

### Plasma and serum folic acid quantification

Plasma and serum folate concentrations were measured using an IMMULITE 2000 Folic Acid assay (Siemens).

### Quantification of folate derivatives by LC-MS

Samples (50 mg liver, or pellets comprising approximately 5 million bone marrow cells) were extracted using 80% methanol containing 25 mM sodium ascorbate and stored at –80 °C for at least 1 h. After thawing, samples were centrifuged, and the supernatant was collected and dried under vacuum. The dried folate extract was reconstituted in a buffer containing 25 mM sodium ascorbate, 50 mM Tris-HCl (pH 7.5), and 0.5% 2-mercaptoethanol, and then treated with γ-glutamyl hydrolase (GGH, 0.23 ug/ul) at 37 °C for 1 h.

Solid-phase extraction (SPE) was performed using Bond Elut-PH columns (Agilent, Part No. 12102062), which were pre-conditioned with 1 mL methanol followed by 1 mL wash buffer (30 mM ascorbic acid in 25 mM ammonium acetate, pH 4.0). Samples were acidified to pH 4.0 with formic acid and loaded onto the conditioned columns. After washing with 1 mL wash buffer, analytes were eluted with 300–400 µL of elution buffer (50:50 water:methanol containing 0.5% 2-mercaptoethanol and 25 mM ammonium acetate, pH 7.0). Eluates were dried under vacuum and reconstituted in water for subsequent mass spectrometry analysis. 1.2 ng of 5-methyl-THF ^13^C_5_ was spiked into each sample as an internal standard.

Chromatographic separation was performed on a Vanquish UHPLC system with a Cadenza CD-C18 3 µm packing column (Imtakt, 2.1 mm id × 150 mm) coupled to a Q Exactive Orbitrap mass spectrometer (Thermo Scientific) via an Ion Max ion source with a HESI II probe (Thermo Scientific). The mobile phase consisted of buffer A: 0.1% formic acid and buffer B: acetonitrile, with 0.1% formic acid. The LC gradient was as follows: 0–2 min, 5% buffer B; 2–7 min, 5–36% buffer B; 7–9 min, 36–95% buffer B; keep 95% buffer B for 2 min; followed by 7 min of re-equilibration of the column before the next run. The flow rate was 150 μL/min. The Q Exactive was operated in full scan, positive mode with the following parameters: the spray voltage 2.5 kV, the heated capillary temperature 300 °C, the HESI probe temperature 350 °C, the sheath gas flow 25 units, the auxiliary gas flow 6 units. MS data acquisition was performed in the m/z range of 440–480, with 70,000 resolution (at 200 m/z). The AGC target was 3e6, and the maximum injection time was 250 ms. The MS data were processed using XCalibur 4.1 (Thermo Scientific) to obtain compound signal intensities.

Samples were normalized based on the total protein in the pellet from each sample that remained after methanol extraction. 0.2 M NaOH (liver: 500 µL, bone marrow: 200 µL) was added to each tissue pellet, and these samples were heated for 20 min at 95 °C with frequent vortexing. The dissolved proteinaceous solution was spun down at 14,000 × *g* for 10 min to remove debris. A DC protein assay (Bio-Rad) was conducted on the supernatant to quantify protein. For analysis, THF/5,10-methylene-THF and 10-formyl-THF/5,10-methenyl-THF were pooled due to the spontaneous interconversion which can occur during sample preparation (Chen et al, 2017; Horne, 2001; Quinlivan et al, 2006).

### Quantification of N2-Me-dG adducts by LC-MS

Organs were snap frozen and stored at –80 °C prior to analysis. DNA was extracted from 10–20 mg tissue chunks using the Qiagen Puregene DNA Extraction kit, homogenizing using a Qiagen Tissuelyzer II. Following isopropanol precipitation of DNA, and washing using 70% ethanol, DNA pellets were resuspended in 500 μl of a 200 mM NaCNBH_3_ solution adjusted to pH 5.2 using a 3 M sodium acetate solution. The samples were left to react at 37 °C, 600 rpm for 24 h to allow reduction of *N2-*hydroxymethyldeoxyguanosine DNA adducts to *N2-*methyldeoxyguanosine (*N2*-Me-dG). Reduced DNA was precipitated using isopropanol, the pellet was washed using 70% ethanol and before resuspension in 80 μl of LC-MS water (Fisher Scientific). DNA concentration was quantified by Nanodrop (Thermo Fisher).

About 10 μg of DNA was digested for 18 h at 37 ^o^C using 0.125 U phosphodiesterase I (Sigma-Aldrich), 0.5 U shrimp alkaline phosphatase (New England Biolabs), and 1 U DNase I (Roche) in a total volume of 100 μl 1X DNAse I buffer (Roche). Two isotopically labeled internal standards (15 fmol [^15^N_5_]-N^2^-methyl-dG, 0.2 nmol [^15^N_5_]- 2-deoxyadenosine) were spiked into the digest. After digestion, samples were filtered using a 3 kDa MWCO PES filter membrane (Analytical), centrifuging at 14,000 × *g* for 20 min.

LC separation of the nucleosides was conducted using a SCIEX M5 microLC trap-elute system (SCIEX). The LC was fitted with a Luna C18(2) trap column (5 µm particle size, 100 Å, 20 mm × 0.50 mm, Phenomenex) and a Kinetex XB-C18 analytical column (2.6 µm particle size, 100 Å, 50 mm × 0.3 mm, Phenomenex) held at 30 °C. The mobile phase conditions consisted of: Solvent A: H_2_O/0.1% acetic acid; Solvent B: ACN/0.1% acetic acid. About 5 μl of digested DNA (500 ng of digested DNA) was injected into a 5 μl sample loop before being loaded onto the trap column using a constant flow rate of 50 μl/min of 0% B for 0.7 min. The nucleosides were eluted onto the analytical column at a constant flow rate of 5 μl/min with an isocratic step of 1% B for 2.8 min, followed by a linear gradient of 1–99% B in 6.4 min. This was followed by three wash pulses of 99–1% B over 3.5 min and equilibration to 1% B for 5.3 min.

MS analysis was conducted using a SCIEX Triple Quad 7500 LC-MS/MS System–QTRAP Ready (SCIEX) interfaced with the LC using an OptiFlow Pro Ion Source (SCIEX). The ion source conditions were as follows: source voltage of 3.5 kV; source temperature of 400 °C; curtain gas at 40 psi; CAD gas at 9 psi; ion source gas 1 at 25 psi; ion source gas 2 at 35 psi. Samples were simultaneously analyzed in both multiple reaction monitoring (MRM/MS^2^) and MS^3^ scan modes. In MRM mode, each sample had a dwell time of 10 ms with a 5 ms pause time. In MS^3^ mode, the LIT was set with a Q3 entry barrier of 10 V, with a fixed fill time of 25 ms. Unique secondary ions were generated during a 25 ms excitation time and scanned at 10,000 Da/s with a step size of 0.12 Da/s.

To allow absolute quantification, samples were analyzed alongside a standard curve ranging from 21.3–680 pmol dA;12-5000 atmol *N2*-Me-dG. Corrected ratio responses were plotted and calculated using a linear regression to determine a standard curve equation. Adducts per dG was calculated by converting dA measurements using their relative distributions in the mouse genome.

### Quantification of total tissue folate content using the *Lactobacillus casei* microbiological assay

Quantification of tissue folate was performed using the *Lactobacillus casei* microbiological assay, as previously described (Suh et al, 2000), with minor modifications to the homogenization procedure. About 20 mg tissue (liver, kidney, spleen, and small intestine) was homogenized in 500 µL extraction buffer and homogenized using a Qiagen Tissuelyzer II. The homogenate was centrifuged at 16,000 × *g* for 5 min, and folate extraction and quantification was performed as previously described (Suh et al, 2000), with bacterial growth quantified at 600 nm on an Epoch Microplate Spectrophotometer (Biotek Instruments). Tissue folate concentrations were normalized to protein concentration, as assessed by the Lowry-Bensadoun assay (Lowry et al, 1951).

### Quantification of micronucleated normochromatic erythrocytes by flow cytometry

Quantification of micronucleated normochomatic erythrocytes (MN-NCEs) was performed using a protocol adapted from one previously described (Balmus et al, 2015). About 30 µL blood (collected by cardiac puncture) was diluted in 150 µL PBS, followed by addition of 135 µL of the resulting mixture to 1.5 mL methanol, pre-cooled to −80 °C, to fix. Fixed blood was stored at −80 °C until staining was performed. To stain, 500 µL fixed blood was washed with 3 mL bicarbonate buffer (5.3 mM sodium bicarbonate in 0.9% saline), centrifuging (500×, 4 °C, 5 min) to pellet cells. The resulting pellet was resuspended in 75 µL bicarbonate buffer. 20 µL of the cell solution was mixed with 80 µL of antibody solution (containing 77 µL bicarbonate buffer, 2 µL RNase A (Sigma) and 1 µL FITC anti-CD71 (Biolegend, 113805)), staining at room temperature for 30 min. Following staining, cells were washed using 700 µL bicarbonate buffer, centrifuging (500 × *g*, 4 °C, 5 min) to pellet cells, and resuspended in 500 µL bicarbonate buffer containing 5 µg/mL propidium iodide (Merck). Samples were then analyzed using a Cytek Aurora CS, calculating the frequency of MN-NCEs as a percentage of total normochromatic erythrocytes.

### Western blots

Protein was extracted from cell line or bone marrow pellets comprising 5 million cells, or 20 mg liver tissue, by homogenizing in 400 µL RIPA buffer (Proteintech) supplemented with 250 U/mL Benzonase Nuclease (Sigma) and Protease/Phosphatase inhibitor (Cell Signaling Technology). Homogenized samples were incubated for 15 min on ice, before centrifuging at 20,000 × *g* for 20 min at 4 °C to pellet debris. Protein in the resulting supernatant was quantified using a Pierce BCA Protein Assay kit (Thermo Scientific). Laemmli buffer (Bio-Rad) supplemented with 2-mercaptoethanol (Sigma) was added to protein samples, followed by boiling at 95 °C for 10 min. About 10 µg protein was loaded in 4–12% Bis-Tris polyacrylamide gels (Genscript) alongside PageRuler Prestained Protein Ladder (Thermo Scientific) and electrophoresed at 120 V for 90 min using MOPS-SDS running buffer (National Diagnostics). Protein was transferred to a 0.45 µm PVDF membrane (GVS), using a standard wet transfer protocol at 35 V for 90 min, with Tris/Glycine transfer buffer (Bio-Rad). The PVDF membrane was cut between the 55 kDA and 70 kDA marker. The upper section was blocked in 5% (w/v) milk (Nestlé) in TBS-0.1% Tween 20, whilst the lower section was blocked in 5% (w/v) BSA (VWR), each for 60 min at room temperature. Membranes were incubated overnight at 4 °C with the following primary antibodies, diluted in the previously used blocking buffer: upper section in anti-vinculin (Proteintech, 26520-1-AP) diluted 1 in 5000; lower section in anti-phospho histone H2AX (Ser139) (Cell Signaling Technology, 2577) diluted 1 in 1000. Membranes were washed three times for 10 min with TBS-0.1% Tween 20, before incubating with HRP-conjugated secondary antibody (Proteintech SA00001-2) diluted 1 in 5000 in the previously used blocking buffer. Membranes were washed three times for 10 min with TBS-0.1% Tween 20. Proteins were visualized using the chemiluminescent Radiance Plus HRP substrate (Azure Biosystems), and imaging was performed using a ChemiDoc MP (Bio-Rad).

### Statistical analysis

Results are plotted as mean ± standard deviation. Mice were randomly assigned to treatment groups, and no blinding was performed during data collection or analysis. Sample sizes for mouse experiments were based on group sizes shown to be sufficient to detect statistically significant differences in formaldehyde-DNA adduct levels in a previous study (Dingler et al, 2020). Consistent with this, the expected significant elevation of formaldehyde-DNA adducts in *Adh5*^*−/−*^ relative to *Adh*^*+/−*^ tissues was detected at these group sizes (Fig. 1F), confirming adequate sensitivity to resolve adduct differences of comparable magnitude. All replicate numbers (“n”) given in the figure legends reflect biological, rather than technical, replicates. Statistical analyses were performed in GraphPad Prism version 10.1.6 (GraphPad Software). Two groups were compared using the Mann–Whitney test. Multiple groups were compared using either the Kruskal–Wallis test with Dunn’s multiple comparisons test (no assumption of normality) or one-way ANOVA with Šidák’s multiple comparisons test (assumption of normality, assessed by the Kolmogorov–-Smirnov test), as indicated in the figure legends. All tests were two-tailed, and *P* < 0.05 was considered statistically significant.

### Declaration of generative artificial intelligence use

During the final preparation of this manuscript, the authors used the generative artificial intelligence (AI) model Claude Opus 4.8 (Anthropic) to assist with spelling and grammar correction, to enhance clarity of phrasing, and support error-checking of references. Generative AI was not used for experimental conceptualization or design, data analysis, figure generation, or the writing of any complete sections of the research manuscript. An AI tool was used to aid the drafting of summary and lay-audience materials (the lay abstract, synopsis, and “The Paper Explained” summary), which were subsequently reviewed and revised by the authors. All AI-assisted content was reviewed, verified, and approved by the corresponding author, who takes full responsibility for the content of the publication.

## Supplementary information

Appendix

Peer Review File

Source data Fig. 1

Source data Fig. 2

Source data Fig. 3

Source data Fig. 4

Figure EV1 Source Data

Figure EV2 Source Data

Figure EV3 Source Data

Figure EV4 Source Data

Figure EV5 Source Data

Expanded View Figures

## Data Availability

All described data are contained within the manuscript. Raw flow cytometry data were deposited at BioStudies (accession: S-BSST3213). The source data of this paper are collected in the following database record: biostudies:S-SCDT-10_1038-S44321-026-00504-7.
