# Supplementary material for: Excess folate elevates formaldehyde genotoxicity in cell lines but not in mice or humans
Source: EMBO Mol Med. 2026 Aug 12;18(9):3430–47. doi: 10.1038/s44321-026-00504-7 (PMC13562568; doi:10.1038/s44321-026-00504-7)
Supplement: Supplementary file 1 [file 44321_2026_504_MOESM12_ESM.pdf]

Expanded View Figures

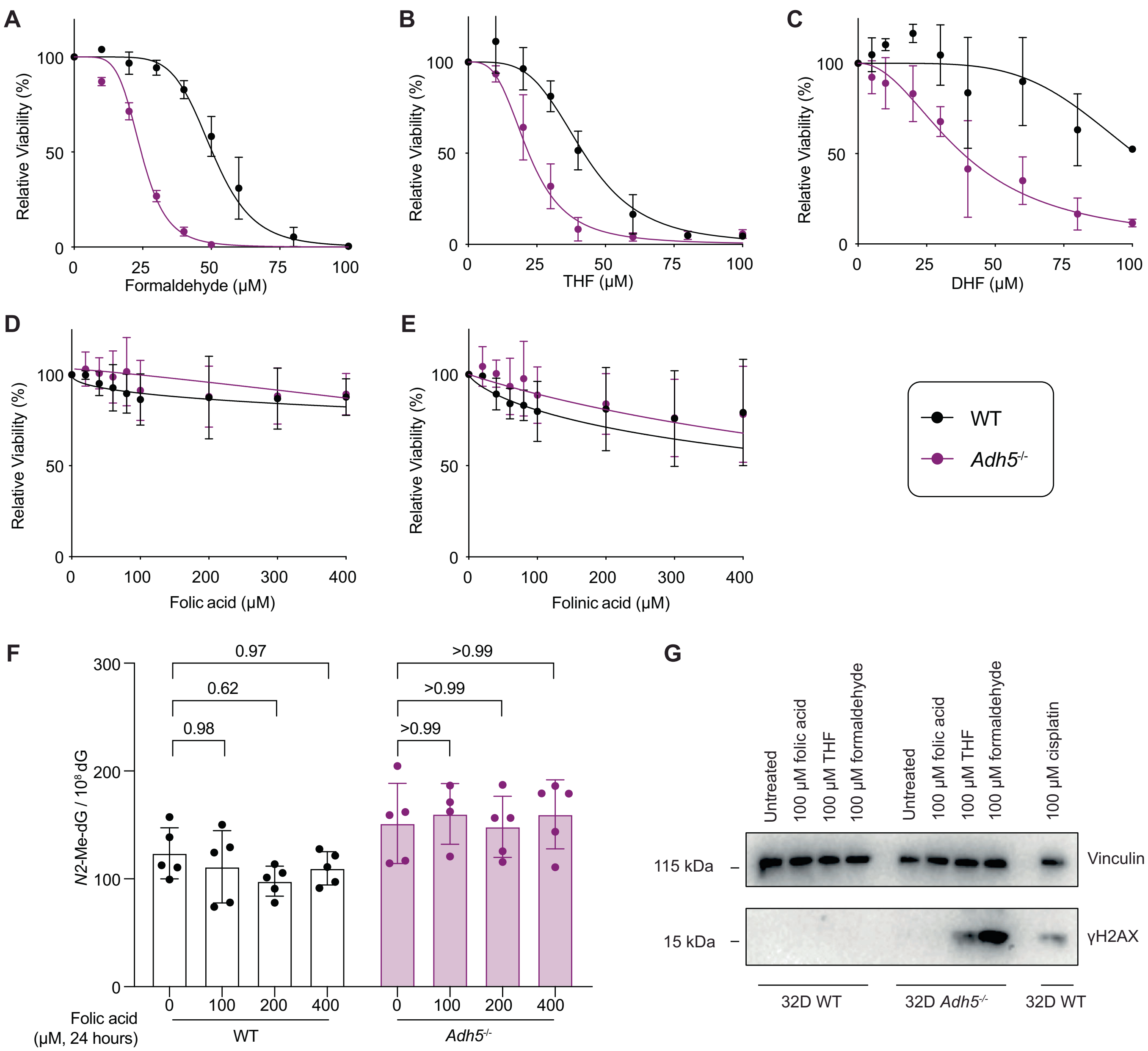

Figure EV1. 32D *Adh5*$^{-/-}$ cells are sensitive to decomposition-prone folate derivatives, but not folic acid.

(**A–E**) Cell survival following 72 h treatment of 32D WT and *Adh5*$^{-/-}$ cells with formaldehyde (**A**), decomposition-prone folates (THF: **B**, DHF: **C**) and decomposition-resistant folates (folic acid: **D**, folinic acid: **E**). (**A**), $n = 2$; (**B–E**), $n = 3$. Mean ± SD. (**F**) Measurement of formaldehyde-induced *N2*-Me-dG adducts by LC-MS in WT and *Adh5*$^{-/-}$ 32D cells, following 24-hour treatment with folic acid. $n = 4$–5, mean ± SD. One-way ANOVA with Šidák multiple comparisons test. (**G**) Western blot showing H2AX serine 139 phosphorylation (γH2AX) in 32D WT and *Adh5*$^{-/-}$ cells following 6-h treatment with folic acid, THF or formaldehyde. 6-h cisplatin treatment was used as an additional positive control. Source data are available online for this figure.

 © The Author(s)

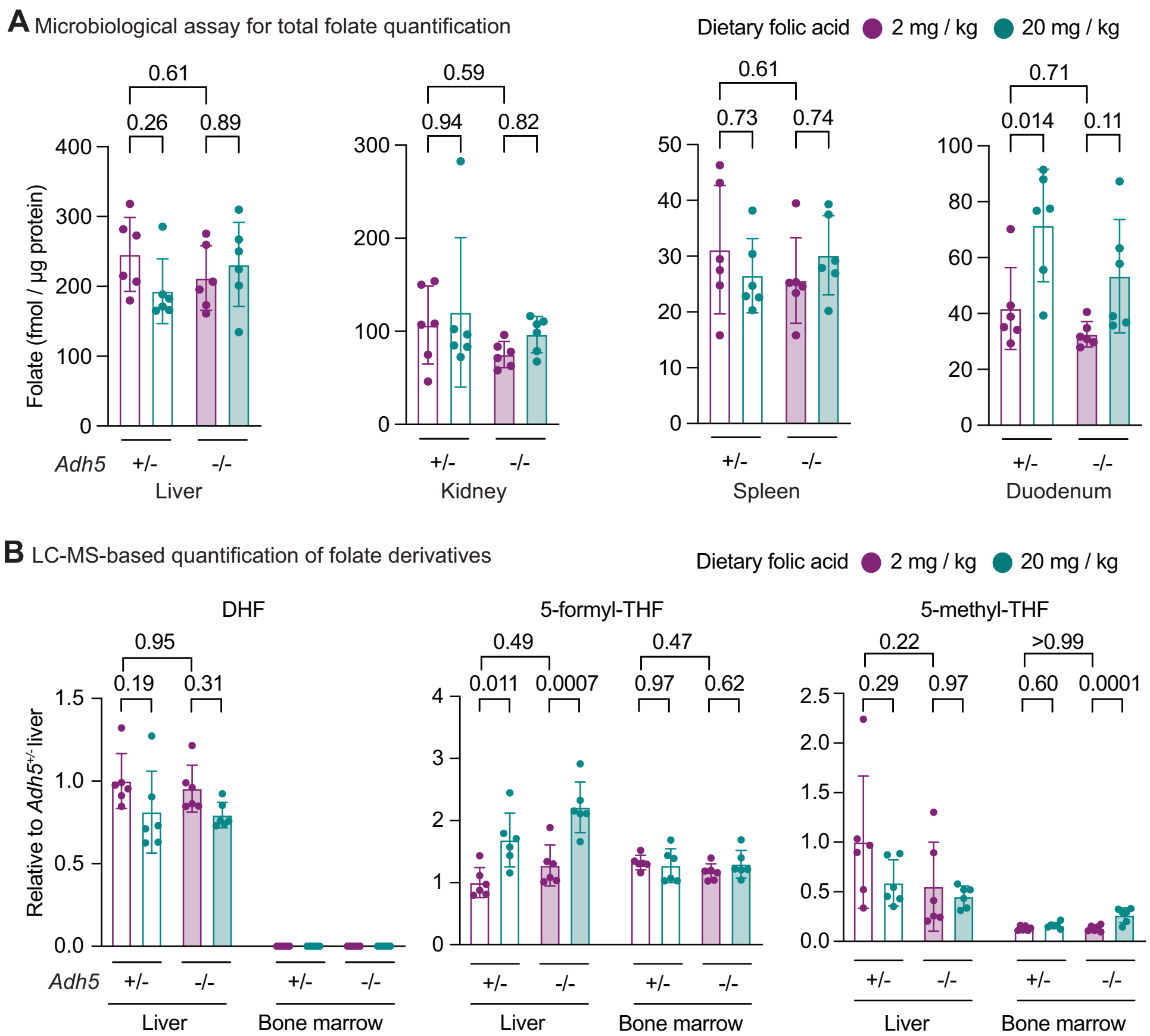

Figure EV2. Quantification of tissue folate content in *Adh5*$^{+/-}$ and *Adh5*$^{-/-}$ mice fed a high-folic acid diet.

(**A**) Total folate concentrations in the liver, kidney, spleen and duodenum of *Adh5*$^{+/-}$ and *Adh5*$^{-/-}$ mice fed a control diet or HFD for 8 weeks, quantified using the *Lactobacillus casei* microbiological assay. $n = 6$, male, mean ± SD. One-way ANOVA with Šidák multiple comparisons test. (**B**) LC-MS quantification of folate derivatives in liver and bone marrow. Values were first normalized to the protein content of each tissue sample, then expressed relative to the corresponding folate derivative in *Adh5*$^{+/-}$ liver. $n = 6$, male, mean ± SD. One-way ANOVA with Šidák multiple comparisons test. Source data are available online for this figure.

© The Author(s)

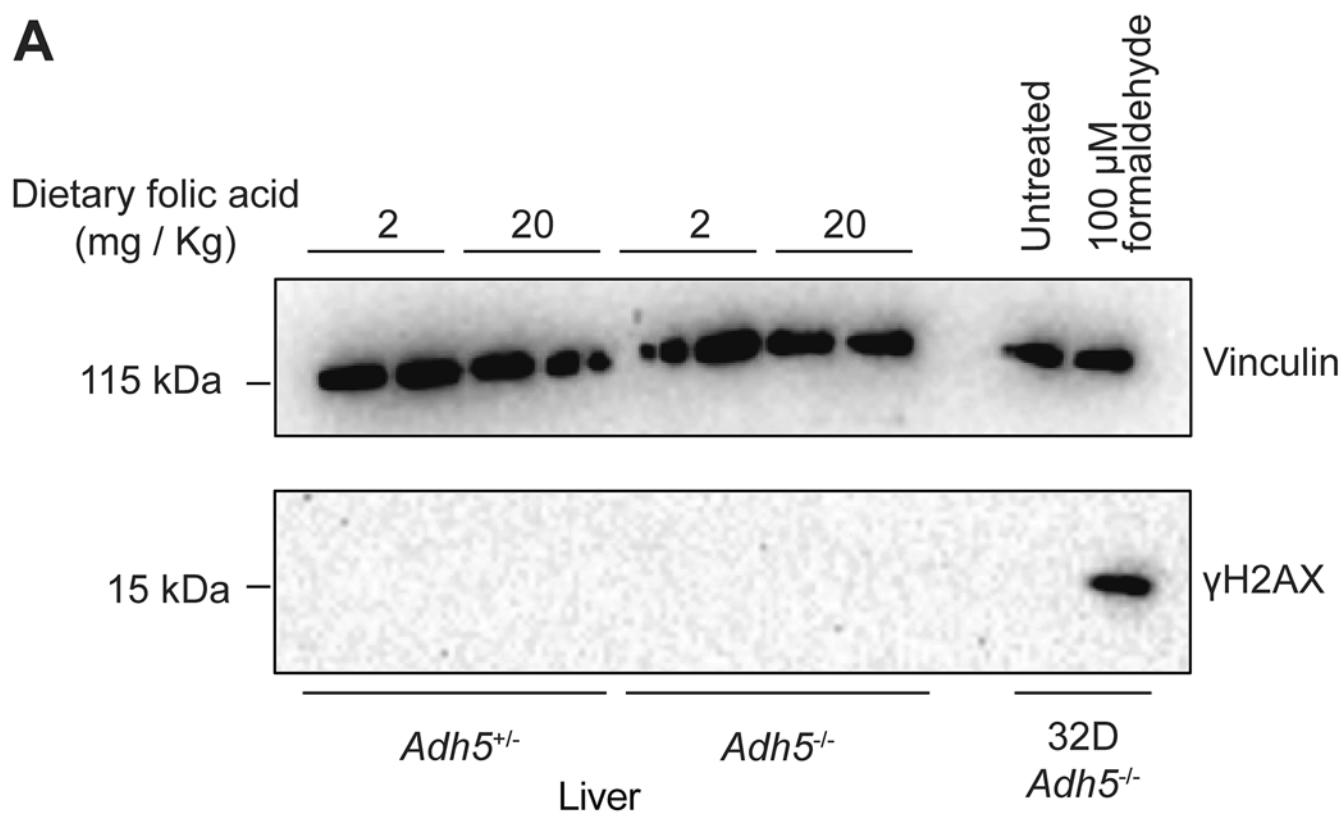

Figure EV3. No detectable γH2AX signal in tissues of *Adh5*$^{-/-}$ mice fed a high-folic acid diet.

Western blots showing no detectable H2AX serine 139 phosphorylation (γH2AX) in the liver (**A**) and bone marrow (**B**) of 11-week-old male *Adh5*$^{+/-}$ and *Adh5*$^{-/-}$ mice fed a control diet or HFD for 8 weeks. 32D *Adh5*$^{-/-}$ cells treated with 100 μM formaldehyde for 6 h were used as a positive control. Source data are available online for this figure.

 © The Author(s)

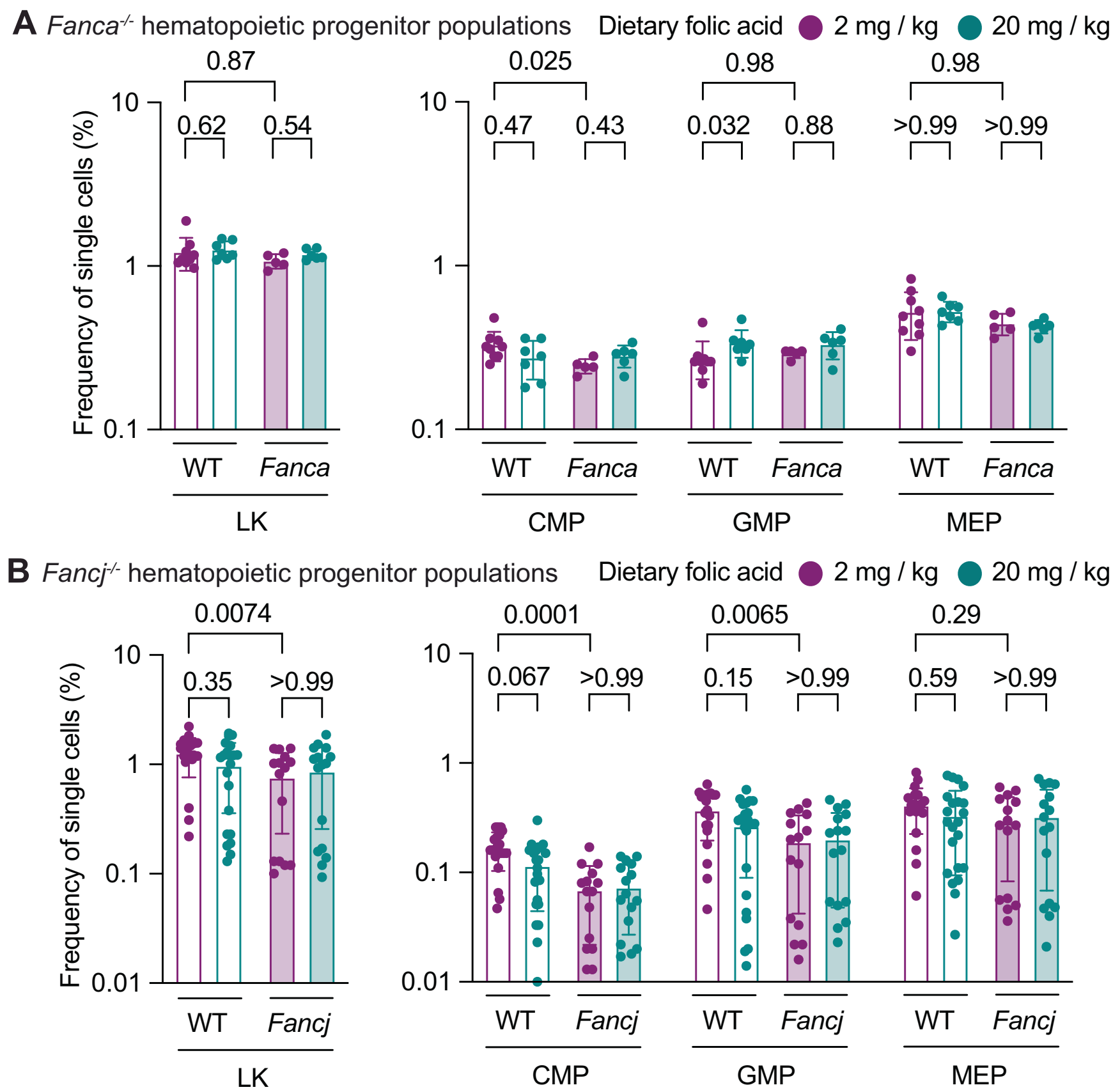

Figure EV4. No change in hematopoietic progenitor frequencies in Fanconi anemia mouse models fed a high-folic acid diet.

Bone marrow progenitor frequencies in 129/S_4 *Fanca*$^{-/-}$ and C57BL/6 J *Fancj*$^{-/-}$ mice fed a HFD for 8 weeks, assessed by flow cytometry. (**A**) Quantification of hematopoietic progenitor populations in *Fanca*$^{-/-}$ mice. 5 = 9, 7, 5, 6 left-to-right. (**B**) Quantification of hematopoietic progenitor populations in *Fancj*$^{-/-}$ mice. $n = 20$, 22, 15, 16 left-to-right. All plots represent merged data from both sexes, mean ± SD. Kruskal–Wallis test with Dunn multiple comparisons test. Source data are available online for this figure.

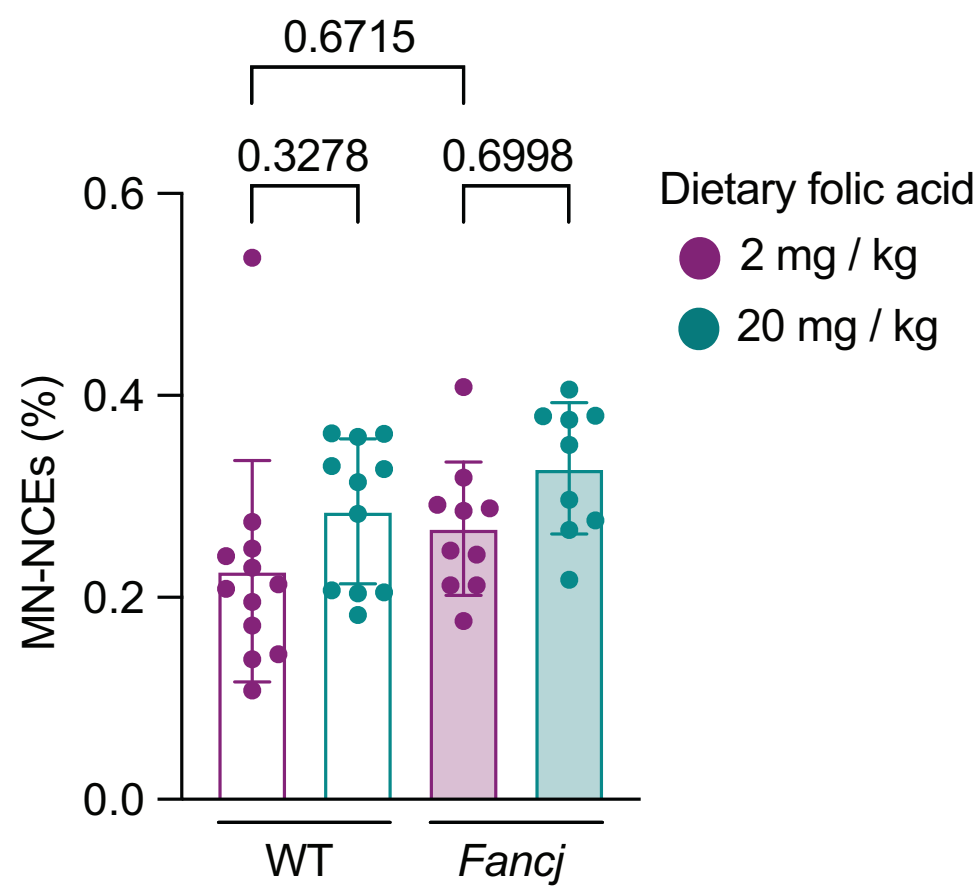

Figure EV5. No change in micronucleated normochromatic erythrocytes in *Fancj*$^{-/-}$ mice fed a high-folic acid diet.

The abundance of MN-NCE, as a percentage of total NCE (MN-NCE + NCE), in C57BL/6 J *Fancj*$^{-/-}$ mice challenged with HFD for 8 weeks. $n = 12, 11, 10, 9$ left-to-right, merged data from both sexes, mean ± SD. Kruskal–Wallis test with Dunn multiple comparisons test. Source data are available online for this figure.

© The Author(s)
