# Supplementary material for: Excess folate elevates formaldehyde genotoxicity in cell lines but not in mice or humans
Source: EMBO Mol Med. 2026 Aug 12;18(9):3430–47. doi: 10.1038/s44321-026-00504-7 (PMC13562568; doi:10.1038/s44321-026-00504-7)
Supplement: Supplementary file 2 [file 44321_2026_504_MOESM1_ESM.pdf]

Excess folate elevates formaldehyde genotoxicity in cell lines but not in mice or humans

Appendix

Table of Contents

Appendix Table S1 2

Appendix Figure S1 3

Appendix Figure S2 4

Appendix Figure S3 5

Appendix Table S1: Demographic data of patients analyzed in Figure 4

Patient ID	Diagnosis	Daily folic acid (mg)	Age	Sex	Plasma folic acid (ng / mL)	PBMC adducts (*N2*-Me-dG / 10^8 dG)
Control 1	Breast Cancer	Control	71	F	13.3	103.91
Control 2	Esophangeal Cancer	Control	81	F	16.7	116.30
Control 3	Breast Cancer	Control	74	F	3.1	109.44
Control 4	Breast Cancer	Control	78	F	3.1	109.94
Control 5	Breast Cancer	Control	52	F	3.8	105.70
Control 6	Cervical Cancer	Control	49	F	5.2	140.08
Control 7	Breast Cancer	Control	49	F	6.4	126.96
Control 8	Breast Cancer	Control	66	F	0.0	132.02
Control 9	Prostate Cancer	Control	75	M	13.6	94.95
Control 10	Prostate Cancer	Control	82	M	5.6	131.43
Control 11	NSCLC	Control	72	M	5.6	115.51
Control 12	Renal Cell Carcinoma	Control	53	M	0.0	60.03
Control 13	Testicular Cancer	Control	41	M	6.6	65.06
Control 14	Hypopharyngeal Cancer	Control	67	M	7.0	130.52
Control 15	Colon Cancer	Control	56	M	11.2	86.59
Folic acid 1	CLL	2	62	M	8.3	134.67
Folic acid 2	Multiple myeloma + Prostate cancer	0.4	75	M	11.3	101.98
Folic acid 3	Marginal Zone Lymphoma	1	76	F	20.1	84.57
Folic acid 4	Colon Cancer	1	72	F	12.0	151.27
Folic acid 5	NSCLC	1	69	F	19.3	82.62
Folic acid 6	Bladder Cancer	1	60	M	17.8	140.98
Folic acid 7	Multiple Myeloma	1	87	M	18.5	87.77
Folic acid 8	Multiple Myeloma	1	90	M	45.2	113.29
Folic acid 9	Prostate Cancer	1	88	M	14.1	100.05

Appendix Figure S1

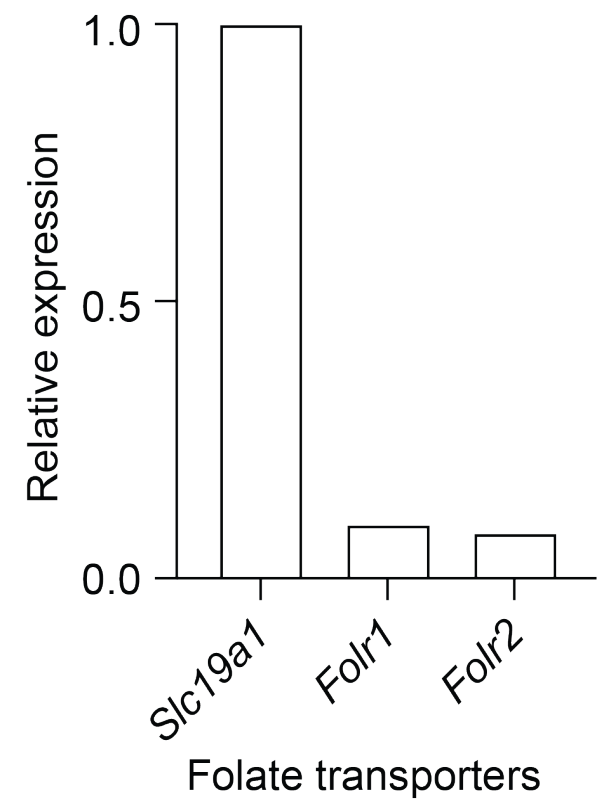

Appendix Figure S1: Relative expression of folate receptors in 32D cells

Relative expression of folate receptors, normalized to *Slc19a1*, as assessed by RNAseq of 32D cells. Data replotted from a previously published RNAseq dataset (Yiqin *et al.*, 2025).

Appendix Figure S2

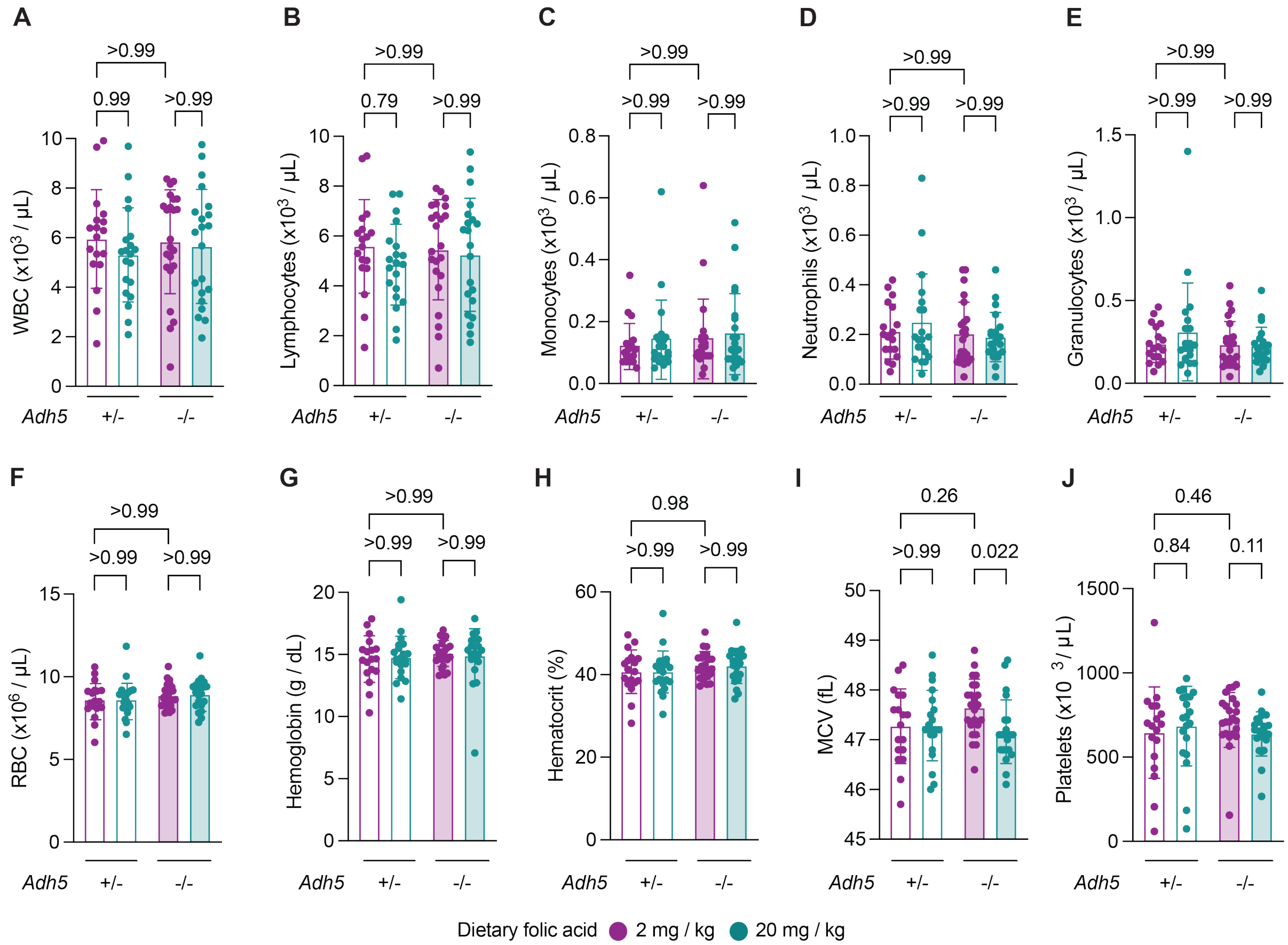

Appendix Figure S2: No change in peripheral blood counts in *Adh5*$^{+/-}$ and *Adh5*$^{-/-}$ mice fed a high folic acid diet

Blood parameters in 11-week-old *Adh5*$^{+/-}$ and *Adh5*$^{-/-}$ mice, following feeding with HFD for 8 weeks, assessed by peripheral blood counts. (A) WBC (white blood cell count). (B) Lymphocytes. (C) Monocytes. (D) Neutrophils. (E) Granulocytes. (F) RBC (red blood cell count). (G) Hemoglobin. (H) Hematocrit. (I) MCV (mean corpuscular volume). (J) Platelets. n=18, 20, 23, 21 left-to-right, mean ± SD. Kruskal-Wallis test with Dunn multiple comparisons test. Male and female mice merged.

Appendix Figure S3

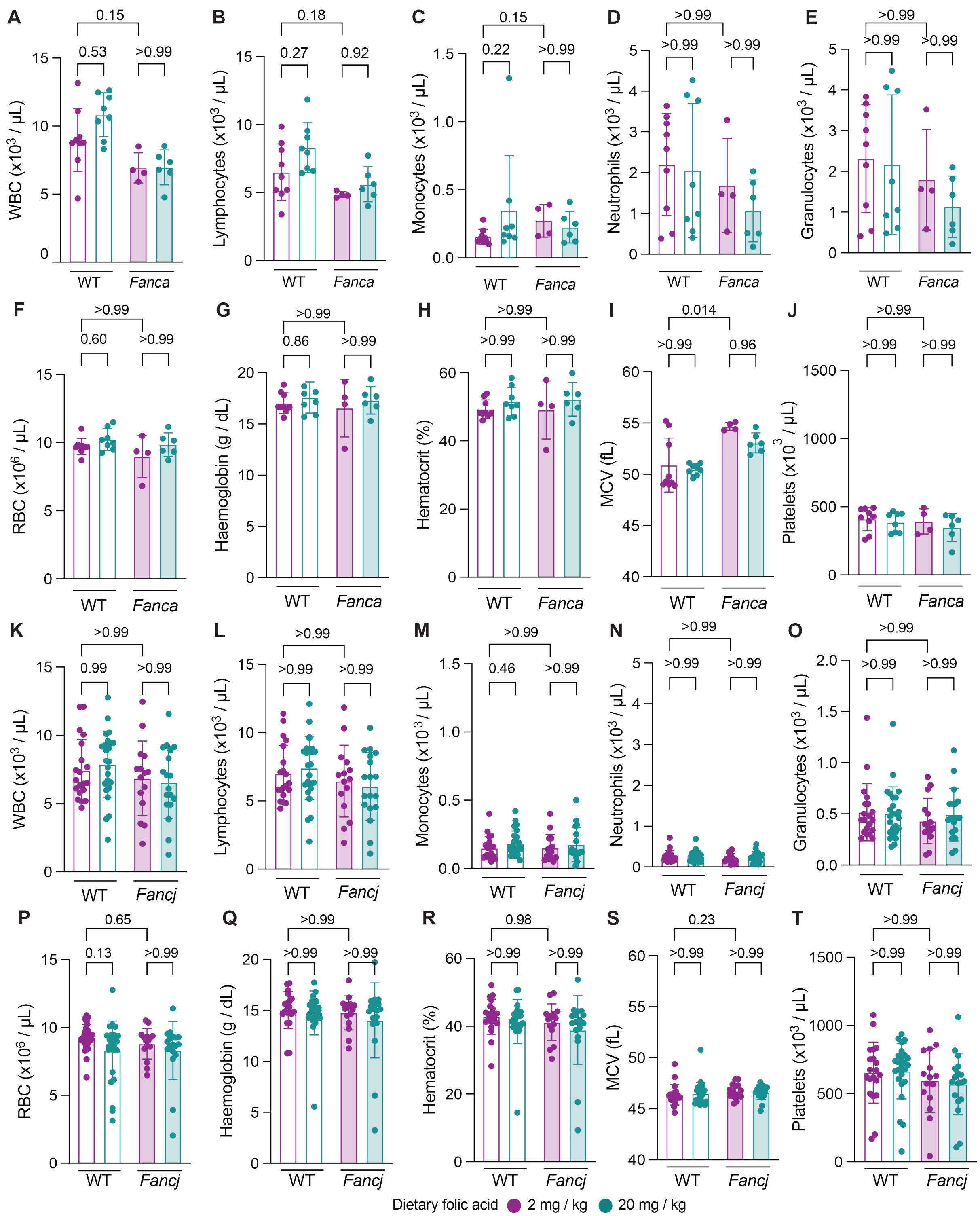

Appendix Figure S3: No change in peripheral blood counts in Fanconi anemia mouse models fed a high folic acid diet

Blood parameters in 11-week-old 129/S_4 *Fanca*$^{-/-}$ and C57BL6/J *Fancj*$^{-/-}$ mice, following feeding with HFD for 8 weeks, assessed by peripheral blood counts. (A, K) WBC (white blood cell count). (B, L) Lymphocytes. (C, M) Monocytes. (D, N) Neutrophils. (E, O) Granulocytes. (F, P) RBC (red blood cell count). (G, Q) Hemoglobin. (H, R) Hematocrit. (I, S) MCV (mean corpuscular volume). (J, T) Platelets. A-J: n=9, 8, 4, 6 left-to-right. K-T: n=20, 25, 15, 18 left-to-right. Mean ± SD. Kruskal-Wallis test with Dunn multiple comparisons test. Male and female mice merged.

References

Yiqin W, Ruimeng Y, Peihong W, Xiaohui L, Hao Y, Hongli H, Yongjian Z et al (2025) Tead1a initiates transcriptional priming through the TEAD1a/YAP-Notch1-Spi1/Cebpα axis to promote neutrophil fate. Adv Sci 12:1–17
