# Supplementary material for: Excess folate elevates formaldehyde genotoxicity in cell lines but not in mice or humans
Source: EMBO Mol Med. 2026 Aug 12;18(9):3430–47. doi: 10.1038/s44321-026-00504-7 (PMC13562568; doi:10.1038/s44321-026-00504-7)
Supplement: Supplementary file 3 [file 44321_2026_504_MOESM2_ESM.pdf]

EMBO Molecular Medicine

Excess folate elevates formaldehyde genotoxicity in cell lines but not in mice or humans

Christopher Mellor, Brandon James, Saeideh Azad, Emma Kosmeder, Chloe Purello, Monika Singh, Victoria Simon, Nicholas Cheng, Jessica Niedermaier, Guillermo Burgos-Barragan, John Blenis, Anthony Mato, Martha Field, and Meng Wang

Corresponding author: Meng Wang (mw997@cornell.edu)

Review Timeline:

Submission Date:	23rd Mar 26
Editorial Decision:	16th Apr 26
Revision Received:	12th Jun 26
Editorial Decision:	9th Jul 26
Revision Received:	26th Jul 26
Accepted:	28th Jul 26

Editor: Lise Roth

Transaction Report:

(Note: With the exception of the correction of typographical or spelling errors that could be a source of ambiguity, letters and reports are not edited. Depending on transfer agreements, referee reports obtained elsewhere may or may not be included in this compilation. Referee reports are anonymous unless the Referee chooses to sign their reports.)

16th Apr 2026

Dear Dr. Wang,

Thank you for submitting your manuscript to our editorial office. We have now received feedback from the three reviewers who agreed to evaluate your study. As you will see in the reports below, the reviewers acknowledge the interest of your findings, but they also recommend that you use more cautious and precise language and clearly state the limitations of your work. The reviewers also suggest a few experiments that would strengthen the conclusions.

We look forward to receiving the revised manuscript according to the referees' recommendations. Please attach a cover letter detailing how you addressed each concern raised by the reviewers. The revised manuscript will be subject to review once again, and we cannot guarantee that the outcome will be favorable at this stage.

If you would like to discuss further the reports from the referees, I am available to do so via email or video. Let me know if you are interested in this option.

We are expecting your revised manuscript within three months, if you anticipate any delay, please contact us.

When preparing your revised manuscript, please refer to our guidelines: https://link.springer.com/journal/44321/submission-guidelines#cms-Revised-submissions. We perform an initial quality control of all revised manuscripts before re-review; failure to include requested items will delay the evaluation of your revision.

We require:

1) A .docx formatted version of the manuscript text (including legends for main figures, EV figures and tables). Please make sure that the changes are highlighted to be clearly visible.

2) Individual production quality figure files as .eps, .tif, .jpg (one file per figure). For guidance, download the 'Figure Guide PDF': https://media.springernature.com/original/springer-cms/rest/v1/content/27825798/data/v1.

3) A .docx formatted letter INCLUDING the reviewers' reports and your detailed point-by-point responses to their comments. As part of the EMBO Press transparent editorial process, the point-by-point response is part of the Review Process File (RPF), which will be published alongside your paper.

4) A complete author checklist, which you can download from our author guidelines. Please insert information in the checklist that is also reflected in the manuscript. The completed author checklist will also be part of the RPF.

5) Please note that all corresponding authors are required to supply an ORCID ID for their name upon submission of a revised manuscript.

6) It is mandatory to include a 'Data Availability' section after the Materials and Methods. Before submitting your revision, primary datasets produced in this study need to be deposited in an appropriate public database, and the accession numbers and database listed under 'Data Availability'. Please remember to provide a reviewer password if the datasets are not yet public.

In case you have no data that requires deposition in a public database, please state so in this section. Note that the Data Availability Section is restricted to new primary data that are part of this study.

7) For data quantification: please specify the name of the statistical test used to generate error bars and P values, the number (n) of independent experiments (specify technical or biological replicates) underlying each data point and the test used to calculate p-values in each figure legend. The figure legends should contain a basic description of n, P and the test applied. Graphs must include a description of the bars and the error bars (s.d., s.e.m.).

8) At EMBO Press we ask authors to provide source data for the main manuscript figures. You will receive a separate email with instructions for providing source data with your revised manuscript, including how to upload and organize the files.

9) Our journal encourages inclusion of *data citations in the reference list* to directly cite datasets that were re-used and obtained from public databases. Data citations in the article text are distinct from normal bibliographical citations and should directly link to the database records from which the data can be accessed. In the main text, data citations are formatted as follows: "Data ref: Smith et al, 2001" or "Data ref: NCBI Sequence Read Archive PRJNA342805, 2017". In the Reference list, data citations must be labeled with "[DATASET]". A data reference must provide the database name, accession number/identifiers and a resolvable link to the landing page from which the data can be accessed at the end of the reference.

10) We replaced Supplementary Information with Expanded View (EV) Figures and Tables that are collapsible/expandable online. A maximum of 5 EV Figures can be typeset. EV Figures should be cited as 'Figure EV1, Figure EV2" etc... in the text and their respective legends should be included in the main text after the legends of regular figures.

- For the figures that you do NOT wish to display as Expanded View figures, they should be bundled together with their legends in a single PDF file called *Appendix*, which should start with a short Table of Content. Appendix figures should be referred to in the main text as: "Appendix Figure S1, Appendix Figure S2" etc.

- Additional Tables/Datasets should be labeled and referred to as Table EV1, Dataset EV1, etc. Legends have to be provided in a separate tab in case of .xls files. Alternatively, the legend can be supplied as a separate text file (README) and zipped together with the Table/Dataset file.

11) The paper explained: EMBO Molecular Medicine articles are accompanied by a summary of the articles to emphasize the major findings in the paper and their medical implications for the non-specialist reader. Please provide a draft summary of your article highlighting
- the medical issue you are addressing,
- the results obtained and
- their clinical impact.

This may be edited to ensure that readers understand the significance and context of the research. Please refer to any of our published articles for an example.

12) Author contributions: You will be asked to provide CRediT (Contributor Role Taxonomy) terms in the submission system. These replace a narrative author contribution section in the manuscript.

13) A "Disclosure and Competing Interests Statement" should be provided in the main text.

14) Every published paper includes a 'Synopsis' to further enhance discoverability. Synopses are displayed on the journal webpage and are freely accessible to all readers. They include a short stand first (maximum of 300 characters, including space) as well as 2-5 one-sentences bullet points that summarizes the paper. Please write the bullet points to summarize the key NEW findings. They should be designed to be complementary to the abstract - i.e. not repeat the same text. We encourage inclusion of key acronyms and quantitative information (maximum of 30 words / bullet point). Please use the passive voice. Please attach these in a separate file or send them by email, we will incorporate them accordingly.

Please also suggest a striking image or visual abstract to illustrate your article as a PNG file 550 px wide x 300 px high.

15) Include a Reagents and Tools Table as part of the Methods section, which can be downloaded from our author guidelines.

16) As part of the EMBO Publications transparent editorial process initiative (see our Editorial at http://embomolmed.embopress.org/content/2/9/329), EMBO Molecular Medicine will publish online a Review Process File (RPF) to accompany accepted manuscripts.
In the event of acceptance, this file will be published in conjunction with your paper and will include the anonymous referee reports, your point-by-point response and all pertinent correspondence relating to the manuscript. Let us know whether you agree with the publication of the RPF and as here, if you want to remove or not any figures from it prior to publication.
Please note that the Authors checklist will be published at the end of the RPF.

EMBO Molecular Medicine has a "scooping protection" policy, whereby similar findings that are published by others during review or revision are not a criterion for rejection. Should you decide to submit a revised version, I do ask that you get in touch after three months if you have not completed it, to update us on the status.

I look forward to receiving your revised manuscript.

Yours sincerely,

Lise Roth

Lise Roth, PhD
Senior Editor
EMBO Molecular Medicine

Please use this link to login to the manuscript system and submit your revision:

***** Reviewer's comments *****

Referee #1 (Comments on Novelty/Model System for Author):

This is a well done study regarding the potential for formaldehyde toxicity from folate, which has never been done before. The use of animal models sensitized to formaldehyde toxicity was particularly elegant.

Referee #1 (Remarks for Author):

In this paper, the authors use a highly sensitive MS method to quantify formaldehyde adducts in cell lines and living animals. After confirming the previously known effect of folate decomposition and formaldehyde production in cultured cells, they next applied their methods to animals in vivo. They used Adh5 and Fanconi anemia knockout mice, which are more sensitive to formaldehyde production and defective in the removal of formaldehyde genotoxicity respectively. The methods used were straightforward. The experiments are properly powered. Figures are well done. Conclusions are warranted.

For this this is a rare manuscript that can be published "as is". I have no suggestions for changes or improvements.

Referee #2 (Remarks for Author):

Mellor et al have performed a study of folate exposure in wildtype mice and mice defective in one of the 2 formaldehyde protection pathways: adh5 or Fanconi anemia pathway. The study follows up on an earlier investigation which found a higher mutagenesis burden in pups born to mice fed a high folate diet. As folate metabolism includes formaldehyde as a metabolic byproduct, the implication was that formaldehyde was causing the high mutagenic signal. This study did not find elevated mutation rates in adult somatic cells, and there were no effects on the viability of HSCs in Adh5-/- or Fanca-/- mice. This suggests that high folate does not induce DNA damage (or actually N2-me-dG is not increase), but does not explain why the previous study found accumulation of mutations. There is also no exploration of whether there are increased mutations observed in either the WT, Adh5-/- or Fanca-/- mice treated. The measurements are indirect: either N2-me-dG/dG ratios for Adh5 experiments in Figure 1, and stem cell number or micronucleus number for Fanca mice. Both of these are only single timepoint measurements that don't capture the same potential long term alterations that were observed in the Cao et al paper which directly measured mutation accumulation.
The results support the less broad conclusion that formaldehyde can't be seen accumulating in blood, serum or liver or the DNA of those cells, and not even when its normal metabolism is blocked. As such, the work is suitable for publication if the above concerns are addressed in text. Several other points in the final discussion section should also be toned down. It is not possible for the authors to conclude that human FA patients are fine with high levels of folate. Human and mouse metabolism, and also viability in absence of FA genes is very different, and the experiments conducted here are only performed with a short exposure period of 8 weeks for mice only. And only some cell types are analysed. The oral cavity may be under higher exposure to formate breakdown products, more similar to cell culture cells, than those in the blood where formaldehyde may already be neutralised. This is a site of "danger" for FA patients, who are prone to oral cancers. Moreover, the Cao et al paper did show elevated mutation frequency in pups born to mice fed a high folate diet. The new data presented here does not examine pregnant mice, so it cannot be said to have relevance to pregnant women given folate supplementation.

Referee #3 (Comments on Novelty/Model System for Author):

As detailed below, some experimental controls are lacking (such as validation of folate and THF uptake into the cells in culture, or the physiologically-relevant form of folate in the in vivo experiments) and some conclusions are not fully supported by the data.

Referee #3 (Remarks for Author):

Reviewer's comments for EMM-2026-23699

Mellor et al address the question whether long-term exposure to high levels of folic acid result in formaldehyde genotoxicity. They first test this in cell culture, in Adh5-null murine bone marrow 32D cell line, where they compared the ensuing formaldehyde genotoxicity following folic acid to THF and DHF supplementation. The authors used LC-MS for N2-Me-dG detection as their readout for formaldehyde genotoxicity and found elevation in N2-Me-dG following treatment with formaldehyde (positive control) and 100 uM THF, bit not 100 uM folic acid.

Next the authors moved to in vivo models where they utilized both Adh5- and Fanc(a/j)-KO models to test the formaldehyde genotoxicity. These models were chosen because they are expected to be more sensitive to formaldehyde damage than WT: Adh5 KO lacks a key enzyme in formaldehyde neutralization, and FancA and FancJ are genes involved in the genotoxic stress repair. In these models the authors did not observe elevated N2-Me-dG or reduced fitness of hematopoietic cells. Finally, the authors tested N2-Me-dG levels in cancer patients with and without folic acid supplementation and observed no elevation in N2-Me-dG, and no correlation with folic acid plasma levels.

These findings are important and should be shared with the community, because they have potential clinical impact for the many patients and individuals who consume folic acid at high concentrations. The work suggest that excess folic acid is not inducing this type of genotoxicity in physiologically-relevant systems, indicating that in this case excess folic acid is not introducing a risk. However - it is important to be careful about the language here because the study can't exclude other excess folic-acid induced toxicities.

Below are some suggestions aimed at tightening the conclusions, so they are supported by the data.

1. The terms "long term use" and "chronic" in the context of high folate exposure should be avoided in the manuscript. The mice were fed HFD for 8 weeks and the patients as little as two weeks. While it is hard to state what is "long term" and "chronic" exposure, the human data is clearly far from it and the mouse data is hard to compare. Therefore, suggesting the data reflects on "long term use" or "chronic" is an extrapolation that is not supported by the data.

2. The experiment that was done in culture raises many questions:

- according to the supp information the media that was used is standard RPMI - this media contains 2 uM folic acid but no other forms of folate. These levels of folic acid are considered supraphysiological and I doubt that adding more on top of that will change the levels of folic acid in the cells. This should be addressed in the text as a caveat, if not experimentally by measuring folic acid in the cells following the supplementation of 100 uM (!).
- The authors should address the folate transporter and folate receptor expression on the 32D cells. This data can be found in public gene expression datasets and is key for validating the relevance of supplementation these cells with reduced vs. non-reduced folate forms. For example - if these cells express high levels of SLC19A1 (that has higher affinity to reduced folate) but not any of the folate receptors, it provides an alternative interpretation of the results. This can be addressed experimentally by LC-MS measurement of the various folate forms in the cells, or addressed by text edits to soften the conclusion and make the interpretation tighter.

3. For the in vivo measurement of folate in Figures 1C,D,E: can the authors explain why they measured these folate forms and not the most abundant and physiologically relevant 5-methyl THF?

4. As mentioned above, the authors should clarify that their findings indeed exclude one type of potential genotoxic stress induced by excess of folic acid, but that other toxicities are not addressed and can't be excluded.

Suggestions / minor:

1. Add in the Introduction an explanation about the folate cycle and the fact that THF, DHF, 5,10-methlene THF, and other folate forms constantly cycle and transform from one form into another.

2. Figures 2C-E and 3D-G: to improve clarity and impact of these panels, consider opening abbreviations of the various cell populations and add a scheme of the hematopoietic differentiation

3. An orthogonal test for the genotoxicity - either by gammaH2AX staining / western blot or another DNA damage marker will strengthen the paper.

Referee #1 (Comments on Novelty/Model System for Author):

This is a well done study regarding the potential for formaldehyde toxicity from folate, which has never been done before. The use of animal models sensitized to formaldehyde toxicity was particularly elegant.

Referee #1 (Remarks for Author):

In this paper, the authors use a highly sensitive MS method to quantify formaldehyde adducts in cell lines and living animals. After confirming the previously known effect of folate decomposition and formaldehyde production in cultured cells, they next applied their methods to animals in vivo. They used Adh5 and Fanconi anemia knockout mice, which are more sensitive to formaldehyde production and defective in the removal of formaldehyde genotoxicity respectively. The methods used were straightforward. The experiments are properly powered. Figures are well done. Conclusions are warranted.

For this this is a rare manuscript that can be published "as is". I have no suggestions for changes or improvements.

We thank Referee #1 for their careful reading of the manuscript, and for their positive comments.

Referee #2 (Remarks for Author):

We thank Referee #2 for their careful consideration of our manuscript.

Mellor et al have performed a study of folate exposure in wildtype mice and mice defective in one of the 2 formaldehyde protection pathways: adh5 or Fanconi anemia pathway. The study follows up on an earlier investigation which found a higher mutagenesis burden in pups born to mice fed a high folate diet. As folate metabolism includes formaldehyde as a metabolic byproduct, the implication was that formaldehyde was causing the high mutagenic signal. This study did not find elevated mutation rates in adult somatic cells, and there were no effects on the viability of HSCs in Adh5-/- or Fanca-/- mice. This suggests that high folate does not induce DNA damage (or actually N2-me-dG is not increase), but does not explain why the previous study found accumulation of mutations. There is also no exploration of whether there are increased mutations observed in either the WT, Adh5-/- or Fanca-/- mice treated. The measurements are indirect: either N2-me-dG/dG ratios for Adh5 experiments in Figure 1, and stem cell number or micronucleus number for Fanca mice. Both of these are only single timepoint measurements that don't capture the same potential long term alterations that were observed in the Cao et al paper which directly measured mutation accumulation.

The results support the less broad conclusion that formaldehyde can't be seen accumulating in blood, serum or liver or the DNA of those cells, and not even when its normal metabolism is blocked. As such, the work is suitable for publication if the above concerns are addressed in text.

We acknowledge these limitations of our study and have added a specific limitations paragraph in Discussion highlighting the reviewers' comments regarding the duration of high-folate diet challenge, the single timepoint measurement and focusing only on formaldehyde-induced DNA adducts.

We did not examine mutation rates as we observed no evidence of increased formaldehyde arising from high-folate diet. Whilst we see no evidence that folic acid supplementation elevates formaldehyde burden in our model systems, this does not exclude that other forms of genotoxic stress. To reflect this, we have added additional text highlighting the 'Cao et al 2023' paper and additional references highlighting other mechanisms of genotoxicity arising from excess folic acid supplementation.

Lines 194 – 210: *'There are limitations to our study. First, we only focus on formaldehyde-induced genotoxicity with a single timepoint of analysis after 8 weeks of HFD in mice. We therefore cannot exclude that a longer duration on HFD could precipitate formaldehyde-genotoxicity from chronic accumulation of folates, which could*

have additive effects with other mechanisms of genotoxicity associated with excess oral folic acid including oxidative damage, nucleotide imbalance and uracil misincorporation (Alnabbat et al., 2022; Henry et al., 2017; Heyden et al., 2024; Koseki et al., 2020). A previous study (Cao et al., 2023) used a longer HFD challenge (16 weeks) in mice to observe a 1.8-fold elevation in de novo mutation rates in their offsprings. Despite this increased mutagenesis, there was no enrichment for any specific mutation patterns that could be attributed to formaldehyde (Blouin et al., 2025; Dingler et al., 2020; Kucab et al., 2019; Thapa et al., 2022). Second, we did not quantify formaldehyde-adducts in the oral cavity, a frequent site of cancer in Fanconi anemia patients. It will be relevant to assess the genotoxic impact of formaldehyde arising from decomposition of folates in food and/or microbiome through direct mucosal contact in the oral cavity. Third, the experimental systems used here does not model pregnancy, so caution is warranted in translating our findings to folic acid supplementation for pregnant people.'

Several other points in the final discussion section should also be toned down. It is not possible for the authors to conclude that human FA patients are fine with high levels of folate. Human and mouse metabolism, and also viability in absence of FA genes is very different, and the experiments conducted here are only performed with a short exposure period of 8 weeks for mice only.

We acknowledge these limitations, and have toned down the closing sentence in the Abstract, and amended Discussion to restrict applicability to short term folic acid supplementation for Fanconi anemia patients.

Lines 190 – 192: *'This has direct relevance for Fanconi anemia patients requiring short-term high-dose folic acid as supportive treatment during bone marrow transplantation or chemotherapy.'*

And only some cell types are analysed. The oral cavity may be under higher exposure to formate breakdown products, more similar to cell culture cells, than those in the blood where formaldehyde may already be neutralised. This is a site of "danger" for FA patients, who are prone to oral cancers.

We acknowledge this limitation in the limitations paragraph in added to Discussion:

Lines 204 – 208: *'Second, we did not quantify formaldehyde-adducts in the oral cavity, a frequent site of cancer in Fanconi anemia patients. It will be relevant to assess the genotoxic impact of formaldehyde arising from decomposition of folates in food and/or microbiome through direct mucosal contact in the oral cavity.'*

Moreover, the Cao et al paper did show elevated mutation frequency in pups born to mice fed a high folate diet. The new data presented here does not examine pregnant

mice, so it cannot be said to have relevance to pregnant women given folate supplementation.

We acknowledge this limitation in the limitations paragraph in added to Discussion:

Lines 208 – 210: *‘Third, the experimental systems used here does not model pregnancy, so caution is warranted in translating our findings to folic acid supplementation for pregnant people.*

Referee #3 (Comments on Novelty/Model System for Author):

As detailed below, some experimental controls are lacking (such as validation of folate and THF uptake into the cells in culture, or the physiologically-relevant form of folate in the in vivo experiments) and some conclusions are not fully supported by the data.

Referee #3 (Remarks for Author):

Reviewer's comments for EMM-2026-23699

Mellor et al address the question whether long-term exposure to high levels of folic acid result in formaldehyde genotoxicity. They first test this in cell culture, in Adh5-null murine bone marrow 32D cell line, where they compared the ensuing formaldehyde genotoxicity following folic acid to THF and DHF supplementation. The authors used LC-MS for N2-Me-dG detection as their readout for formaldehyde genotoxicity and found elevation in N2-Me-dG following treatment with formaldehyde (positive control) and 100 uM THF, bit not 100 uM folic acid.
Next the authors moved to in vivo models where they utilized both Adh5- and Fanc(a/j)-KO models to test the formaldehyde genotoxicity. These models were chosen because they are expected to be more sensitive to formaldehyde damage than WT: Adh5 KO lacks a key enzyme in formaldehyde neutralization, and FancA and FancJ are genes involved in the genotoxic stress repair. In these models the authors did not observe elevated N2-Me-dG or reduced fitness of hematopoietic cells. Finally, the authors tested N2-Me-dG levels in cancer patients with and without folic acid supplementation and observed no elevation in N2-Me-dG, and no correlation with folic acid plasma levels. These findings are important and should be shared with the community, because they have potential clinical impact for the many patients and individuals who consume folic acid at high concentrations. The work suggest that excess folic acid is not inducing this type of genotoxicity in physiologically-relevant systems, indicating that in this case excess folic acid is not introducing a risk. However - it is important to be careful about the language here because the study can't exclude other excess folic-acid induced toxicities.
Below are some suggestions aimed at tightening the conclusions, so they are supported by the data.

We thank Referee #3 for their consideration of our manuscript. We lay out point-by-point the changes requested below.

1. The terms "long term use" and "chronic" in the context of high folate exposure should be avoided in the manuscript. The mice were fed HFD for 8 weeks and the patients as

little as two weeks. While it is hard to state what is "long term" and "chronic" exposure, the human data is clearly far from it and the mouse data is hard to compare. Therefore, suggesting the data reflects on "long term use" or "chronic" is an extrapolation that is not supported by the data.

We have removed use of ‘long-term’ and ‘chronic’ where they previously appeared, on lines 87 and 189.

In addition, we have added a limitations paragraph to Discussion highlighting the 8-week duration of HFD may not model longer ‘chronic’ exposure:

Lines 194 – 200: *‘There are limitations to our study. First, we only focus on formaldehyde-induced genotoxicity with a single timepoint of analysis after 8 weeks of HFD in mice. We therefore cannot exclude that a longer duration on HFD could precipitate formaldehyde-genotoxicity from chronic accumulation of folates, which could have additive effects with other mechanisms of genotoxicity associated with excess oral folic acid including oxidative damage, nucleotide imbalance and uracil misincorporation (Alnabbat et al., 2022; Henry et al., 2017; Heyden et al., 2024; Koseki et al., 2020).’*

Furthermore, as requested by Reviewer #1, we have toned down the relevant text in our discussion to restrict applicability to short term folic acid supplementation for Fanconi anemia patients.

Lines 190 – 192: *‘This has direct relevance for Fanconi anemia patients requiring short-term high-dose folic acid as supportive treatment during bone marrow transplantation or chemotherapy.’*

2. The experiment that was done in culture raises many questions:
- according to the supp information the media that was used is standard RPMI - this media contains 2 uM folic acid but no other forms of folate. These levels of folic acid are considered supraphysiological and I doubt that adding more on top of that will change the levels of folic acid in the cells. This should be addressed in the text as a caveat, if not experimentally by measuring folic acid in the cells following the supplementation of 100 uM (!).
- The authors should address the folate transporter and folate receptor expression on the 32D cells. This data can be found in public gene expression datasets and is key for validating the relevance of supplementation these cells with reduced vs. non-reduced folate forms. For example - if these cells express high levels of SLC19A1 (that has higher affinity to reduced folate) but not any of the folate receptors, it provides an alternative interpretation of the results. This can be addressed experimentally by LC-MS measurement of the various folate forms in the cells, or addressed by text edits to soften the conclusion and make the interpretation tighter.

Thank you for making this insightful suggestion. We have analyzed the expression of folate receptors and *Slc19a1* using a published RNAseq dataset from 32D cells. 32D cells indeed express significantly lower levels of folate transporters (Folr1/Folr2) compared to *Slc19a*. We have added this figure to the Appendix (Appendix Figure 1).

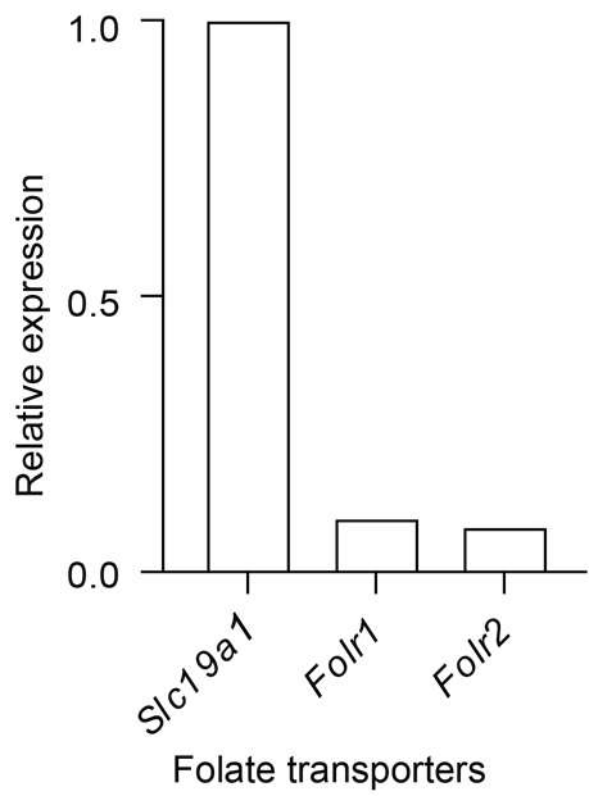

This limitation of cell culture model further validates our approach of using in vivo whole-organisms to model the effect of excess oral folic acid intake on endogenous formaldehyde. Since we already have results from both mouse and human, measuring folate metabolites in the 32D cell line would ultimately not change the conclusions drawn from mouse and human. We have therefore amended the text in Results accordingly:

Lines 118 – 126: *'A possible explanation for the absence of elevated endogenous formaldehyde–DNA adducts in 32D cells under supraphysiological folic acid supplementation is that folic acid uptake is saturated. Consistent with this, 32D cells express 10-fold lower levels of folate receptors (Folr1, Folr2), which preferentially take up folic acid compared to SLC19A1 (**Appendix Figure 1**), which exhibits preference to carry reduced folates (Zhao et al., 2009). Consequently, to overcome this limitation of the cell culture model, we proceeded to model excess oral folic acid intake in mice to test the contribution of physiological gastrointestinal absorption of folic acid and its conversion to decomposition-prone reduced folates as a source of endogenous formaldehyde.'*

3. For the in vivo measurement of folate in Figures 1C,D,E: can the authors explain why they measured these folate forms and not the most abundant and physiologically relevant 5-methyl THF?

Measurement of other folate forms are included in Extended View Figure 2 (which we have now promoted from the appendix, to an extended view figure, to draw greater attention to their presence).

4. As mentioned above, the authors should clarify that their findings indeed exclude one type of potential genotoxic stress induced by excess of folic acid, but that other toxicities are not addressed and can't be excluded.

We agree. This is addressed with added text in the limitations paragraph in Discussion lines 194 – 200 as inserted for point 1,

Suggestions / minor:

1. Add in the Introduction an explanation about the folate cycle and the fact that THF, DHF, 5,10-methlene THF, and other folate forms constantly cycle and transform from one form into another.

Thank you, we agree that this is needed to introduce the folate cycle. We have added to Introduction an explanation on the folate cycle and the interconversion of folate metabolites:

Lines 76 – 84 *'There is a direct biochemical link between folates and formaldehyde. Following absorption, folic acid undergoes stepwise enzymatic reduction to dihydrofolate (DHF) and tetrahydrofolate (THF), which receives and shuttles one-carbon units through a series of cyclic enzymatic interconversions between 5,10-methylene-THF, 5,10-methenyl-THF and 10-formyl-THF to support thymidine and purines synthesis. In addition, the irreversible conversion of 5,10-methylene-THF to 5-methyl-THF provides the one-carbon unit for methionine synthesis, supporting formation of the universal methyl donor S-adenosylmethionine and, via transsulfuration of homocysteine, the redox regulator glutathione (Ducker and Rabinowitz, 2017; Fox and Stover, 2008).'*

2. Figures 2C-E and 3D-G: to improve clarity and impact of these panels, consider opening abbreviations of the various cell populations and add a scheme of the hematopoietic differentiation

We have added a panel demonstrating the differentiation hierarchy of hematopoetic stem and progenitor cells, containing abbreviations of the cell populations, as Figure 2A.

3. An orthogonal test for the genotoxicity - either by gammaH2AX staining / western blot or another DNA damage marker will strengthen the paper.

We have now performed Western blots assessing γH2AX (Ser139) levels for both cell line and murine tissues. In cell lines, we observed elevated γH2AX in cells treated with THF but not folic acid (Extended View Figure 1G), consistent with our adductomics results, which show that treatment of *Adh5*$^{-/-}$ cells with THF, but not folic acid, increased

N2-Me-dG adduct levels. In liver and bone marrow from *Adh5*$^{+/-}$ and *Adh5*$^{-/-}$ mice, we observed no induction of γH2AX following HFD (Extended View Figure 3).

We have updated text in Results:

Lines 115 – 118 *'Consistent with previous observations (García-Calderón et al., 2018), THF treatment of Adh5*$^{-/-}$ *32D cells induced serine 139 phosphorylation of H2AX (γH2AX), a cellular marker of DNA damage and repair (**Fig EV1G**). However, this was not observed following treatment with folic acid.'*

Lines 143 – 145 *'Consistent with the lack of elevated adducts, we also observed no induction of γH2AX by immunoblotting in either the liver or bone marrow of Adh5*$^{+/-}$ *or Adh5*$^{-/-}$ *mice fed HFD (**Fig EV3**).'*

The methodology for the Western blot experiment is included in the amended Appendix.

9th Jul 2026

Dear Dr. Wang,

Thank you for submitting your revised manuscript, which has now been evaluated by referees #2 and #3. As you will see below, they are satisfied with the revisions, and I will therefore be able to accept your manuscript once the following editorial points have been addressed:

1/ Manuscript text:
- Please remove the highlighted text and only keep in track changes mode any new modification.
- Methods:
o As we don't have text limitations, please include the supplementary methods in the main manuscript text methods, and update the checklist accordingly.
o Animals: indicate sex and age of mice for all experiments.
o Patient blood samples: provide the full sentence that the experiments conformed to the principles set out in the WMA Declaration of Helsinki and the Department of Health and Human Services Belmont Report, and provide reference number for study approval.
- Data availability: please remove the current text and list here datasets generated as part of this study. If no dataset that requires deposition was generated, indicate: "This study includes no data deposited in external repositories".
- The disclosure and competing interest statement should be placed after the Acknowledgements.
- Remove Authors contribution and provide CRediT (Contributor Role Taxonomy) terms in the submission system instead. These replace a narrative author contribution section in the manuscript.

2/ Figures:
- Please correct the nomenclature of EV figures to Figure EV1, etc.
- Appendix: remove the authors from the file, and move the methods and references to the main manuscript text. The PDF needs to be clean (without highlighting, track changes, etc.). The separate Appendix Figures should be removed as we only need them in the Appendix file.
- The callout for Appendix Table 1 in the text should be corrected to Appendix Table S1.

3/ Author checklist: please fill in the section Experimental study design and statistics, subsection Inclusion/exclusion criteria, as you have provided this information in the manuscript (patients).

4/ Source Data: please also provide raw facs data.

5/ Synopsis and Paper Explained:
- Please upload your synopsis text as an individual file.
- Please resize your visual abtract to 550 px wide x 300 px high, and make sure that the text remains legible.
- Please integrate the Paper Explained in the main manuscript file, after the introduction.
- The lay summary is not needed.

6/ As part of the EMBO Publications transparent editorial process initiative (see our Editorial at http://embomolmed.embopress.org/content/2/9/329), EMBO Molecular Medicine will publish online a Review Process File (RPF) to accompany accepted manuscripts.
This file will be published in conjunction with your paper and will include the anonymous referee reports, your point-by-point response and all pertinent correspondence relating to the manuscript. Let us know whether you agree with the publication of the RPF and as here, if you want to remove or not any figures from it prior to publication.
Please note that the Authors checklist will be published at the end of the RPF.

I look forward to receiving your revised manuscript.

Yours sincerely,

Lise Roth

Lise Roth, PhD
Senior Editor
EMBO Molecular Medicine

***** Reviewer's comments *****

Referee #2 (Remarks for Author):

The authors have addressed my concerns

Referee #3 (Comments on Novelty/Model System for Author):
The authors used several models and cross validated their results between them.
These findings are important and should be shared with the community, because they have potential clinical impact for the many patients and individuals who consume folic acid at high concentrations. The work suggest that excess folic acid is not inducing this type of genotoxicity in physiologically-relevant systems, indicating that in this case excess folic acid is not introducing a risk.

Referee #3 (Remarks for Author):

The authors responded well to the comments and I congratulate them on this paper.

The authors addressed the remaining editorial issues.

28th Jul 2026

Dear Dr. Wang,

Thank you for submitting your revised files. I am pleased to inform you that your manuscript is accepted for publication and is now being sent to our publisher to be included in the next available issue of EMBO Molecular Medicine.

Please note that I have inserted back the following sentence in the Methods (previously in your Appendix):
"Cells tested negative for mycoplasma, and when tested by ATCC, had STR profiles
consistent with the 32D clone 3 reference."
Please let us know immediately if this should be changed.

Your manuscript will be processed for publication by EMBO Press. It will be copy edited and you will receive page proofs prior to publication. Please note that you will be contacted by Springer Nature Author Services to complete licensing and payment information.

You may qualify for financial assistance for your publication charges - either via a Springer Nature fully open access agreement or an EMBO initiative. Check your eligibility: https://link.springer.com/journal/44321/how-to-publish-with-us

Should you be planning a Press Release on your article, please notify the production team via this form in order to coordinate publication and release dates: https://forms.cloud.microsoft/e/eH4A8JVP6g

If you have any questions, please do not hesitate to contact the Editorial Office. Thank you for your contribution to EMBO Molecular Medicine.

Yours sincerely,

Lise Roth

Lise Roth, Ph.D
Senior Editor
EMBO Molecular Medicine

>>> Please note that it is EMBO Molecular Medicine policy for the transcript of the editorial process (containing referee reports and your response letter) to be published as an online supplement to each paper. If you do NOT want this, you will need to inform the Editorial Office via email immediately. More information is available here: https://link.springer.com/partners/embo-press/editorial-policies#Peer%20review

EMBO Press Author Checklist

Corresponding Author Name: Meng Wang	
Journal Submitted to: EMBO Molecular Medicine	
Manuscript Number: EMM-2026-23699	

USEFUL LINKS FOR COMPLETING THIS FORM
The EMBO Journal - Author Guidelines
EMBO Reports - Author Guidelines
Molecular Systems Biology - Author Guidelines
EMBO Molecular Medicine - Author Guidelines

Reporting Checklist for Life Science Articles (updated January
This checklist is adapted from Materials Design Analysis Reporting (MDAR) Checklist for Authors. MDAR establishes a minimum set of requirements in transparent reporting in the life sciences (see Statement of Task: 10.31222/osf.io/9sm4x). Please follow the journal's guidelines in preparing your
Please note that a copy of this checklist will be published alongside your article.

Abridged guidelines for figures
1. Data
The data shown in figures should satisfy the following conditions:
- the data were obtained and processed according to the field's best practice and are presented to reflect the results of the experiments in an accurate and unbiased manner.
- ideally, figure panels should include only measurements that are directly comparable to each other and obtained with the same assay.
- plots include clearly labeled error bars for independent experiments and sample sizes. Unless justified, error bars should not be shown for technical
- if n<5, the individual data points from each experiment should be plotted. Any statistical test employed should be justified.
- Source Data should be included to report the data underlying figures according to the guidelines set out in the authorship guidelines on Data

2. Captions
Each figure caption should contain the following information, for each panel where they are relevant:
- a specification of the experimental system investigated (eg cell line, species name).
- the assay(s) and method(s) used to carry out the reported observations and measurements.
- an explicit mention of the biological and chemical entity(ies) that are being measured.
- an explicit mention of the biological and chemical entity(ies) that are altered/varied/perturbed in a controlled manner.
- the exact sample size (n) for each experimental group/condition, given as a number, not a range;
- a description of the sample collection allowing the reader to understand whether the samples represent technical or biological replicates (including how many animals, litters, cultures, etc.).
- a statement of how many times the experiment shown was independently replicated in the laboratory.
- definitions of statistical methods and measures:
  - common tests, such as t-test (please specify whether paired vs. unpaired), simple χ2 tests, Wilcoxon and Mann-Whitney tests, can be unambiguously identified by name only, but more complex techniques should be described in the methods section;
  - are tests one-sided or two-sided?
  - are there adjustments for multiple comparisons?
  - exact statistical test results, e.g., P values = x but not P values < x;
  - definition of 'center values' as median or average;
  - definition of error bars as s.d. or s.e.m.

Please complete ALL of the questions below.
Select "Not Applicable" only when the requested information is not relevant for your study.

Materials

Newly Created Materials	Information included in the manuscript?	In which section is the information available? (Reagents and Tools Table, Materials and Methods, Figures, Data Availability Section)
New materials and reagents need to be available; do any restrictions apply?	Not Applicable	

Antibodies	Information included in the manuscript?	In which section is the information available? (Reagents and Tools Table, Materials and Methods, Figures, Data Availability Section)
For **antibodies** provide the following information:<br>- Commercial antibodies: RRID (if possible) or supplier name, catalogue number and or/clone number<br>- Non-commercial: RRID or citation	Yes	Reagents and Tools Table

DNA and RNA sequences	Information included in the manuscript?	In which section is the information available? (Reagents and Tools Table, Materials and Methods, Figures, Data Availability Section)
**Short novel DNA or RNA including primers, probes**: provide the sequences.	Not Applicable	

Cell materials	Information included in the manuscript?	In which section is the information available? (Reagents and Tools Table, Materials and Methods, Figures, Data Availability Section)
**Cell lines:** Provide species information, strain. Provide accession number in repository **OR** supplier name, catalog number, clone number, and/**OR** RRID.	Yes	Reagent and Tools Table
**Primary cultures:** Provide species, strain, sex of origin, genetic modification status.	Not Applicable	
Report if the cell lines were recently **authenticated** (e.g., by STR profiling) and tested for mycoplasma contamination.	Yes	Materials and Methods

Experimental animals	Information included in the manuscript?	In which section is the information available? (Reagents and Tools Table, Materials and Methods, Figures, Data Availability Section)
**Laboratory animals or Model organisms:** Provide species, strain, sex, age, genetic modification status. Provide accession number in repository **OR** supplier name, catalog number, clone number, **OR** RRID.	Yes	Reagent and Tools Table, Materials and Methods
**Animal observed in or captured from the field:** Provide species, sex, and age where possible.	Not Applicable	
Please detail **housing and husbandry conditions**.	Yes	Materials and Methods

Plants and microbes	Information included in the manuscript?	In which section is the information available? (Reagents and Tools Table, Materials and Methods, Figures, Data Availability Section)
**Plants:** provide species and strain, ecotype and cultivar where relevant, unique accession number if available, and source (including location for collected wild specimens).	Not Applicable	
**Microbes:** provide species and strain, unique accession number if available, and source.	Not Applicable	

Human research participants	Information included in the manuscript?	In which section is the information available? (Reagents and Tools Table, Materials and Methods, Figures, Data Availability Section)
If collected and within the bounds of privacy constraints report on age, sex and gender or ethnicity for all study participants.	Yes	Materials and Methods

Core facilities	Information included in the manuscript?	In which section is the information available? (Reagents and Tools Table, Materials and Methods, Figures, Data Availability Section)
If your work benefited from core facilities, was their service mentioned in the acknowledgments section?	Yes	Acknowledgements

Design

Study protocol	Information included in the manuscript?	In which section is the information available? (Reagents and Tools Table, Materials and Methods, Figures, Data Availability Section)
If study protocol has been **pre-registered, provide DOI in the manuscript**. For clinical trials, provide the trial registration number **OR** cite DOI.	Not Applicable	
Report the **clinical trial registration number** (at ClinicalTrials.gov or equivalent), where applicable.	Not Applicable	

Laboratory protocol	Information included in the manuscript?	In which section is the information available? (Reagents and Tools Table, Materials and Methods, Figures, Data Availability Section)
Provide DOI OR other citation details if **external detailed step-by-step protocols** are available.	Yes	References, Materials and Methods

Experimental study design and statistics	Information included in the manuscript?	In which section is the information available? (Reagents and Tools Table, Materials and Methods, Figures, Data Availability Section)
Include a statement about **sample size** estimate even if no statistical methods were used.	Yes	Materials and Methods
Were any steps taken to minimize the effects of subjective bias when allocating animals/samples to treatment (e.g. **randomization procedure**)? If yes, have they been described?	Yes	Materials and Methods
Include a statement about **blinding** even if no blinding was done.	Yes	Materials and Methods
Describe **inclusion/exclusion criteria** if samples or animals were excluded from the analysis. Were the criteria pre-established?<br><br>If sample or data points were omitted from analysis, report if this was due to attrition or intentional exclusion and provide justification.	Yes	Materials and Methods
For every figure, are **statistical tests** justified as appropriate? Do the data meet the assumptions of the tests (e.g., normal distribution)? Describe any methods used to assess it. Is there an estimate of variation within each group of data? Is the variance similar between the groups that are being statistically compared?	Yes	Materials and Methods, Figure Legends

Sample definition and in-laboratory replication	Information included in the manuscript?	In which section is the information available? (Reagents and Tools Table, Materials and Methods, Figures, Data Availability Section)
In the figure legends: state number of times the experiment was **replicated** in laboratory.	Yes	Figure legends
In the figure legends: define whether data describe **technical or biological replicates**.	Yes	Figure legends, Materials and Methods

Ethics

Ethics	Information included in the manuscript?	In which section is the information available? (Reagents and Tools Table, Materials and Methods, Figures, Data Availability Section)
Studies involving **human participants**: State details of **authority granting ethics approval** (IRB or equivalent committee(s), provide reference number for approval.	Yes	Materials and Methods
Studies involving **human participants**: Include a statement confirming that **informed consent** was obtained from all subjects and that the experiments conformed to the principles set out in the WMA Declaration of Helsinki and the Department of Health and Human Services Belmont Report.	Yes	Materials and Methods
Studies involving **human participants:** For publication of **patient photos**, include a statement confirming that consent to publish was obtained.	Not Applicable	
Studies involving experimental **animals**: State details of **authority granting ethics approval** (IRB or equivalent committee(s), provide reference number for approval. Include a statement of compliance with ethical regulations.	Yes	Materials and Methods
Studies involving **specimen and field samples:** State if relevant **permits** obtained, provide details of authority approving study; if none were required, explain why.	Not Applicable	

Dual Use Research of Concern (DURC)	Information included in the manuscript?	In which section is the information available? (Reagents and Tools Table, Materials and Methods, Figures, Data Availability Section)
Could your study fall under dual use research restrictions? Please check biosecurity documents and list of **select agents and toxins** (CDC): https://www.selectagents.gov/sat/list.htm	Not Applicable	
If you used a select agent, is the security level of the lab appropriate and reported in the manuscript?	Not Applicable	
If a study is subject to dual use research of concern regulations, is the name of the **authority granting approval and reference number** for the regulatory approval provided in the manuscript?	Not Applicable	

Reporting

The MDAR framework recommends adoption of discipline-specific guidelines, established and endorsed through community initiatives. Journals have their own policy about requiring specific guidelines and recommendations to complement MDAR.

Adherence to community standards	Information included in the manuscript?	In which section is the information available? (Reagents and Tools Table, Materials and Methods, Figures, Data Availability Section)
State if relevant guidelines or checklists (e.g., **ICMJE, MIBBI, ARRIVE, PRISMA**) have been followed or provided.	Not Applicable	
For **tumor marker prognostic studies,** we recommend that you follow the **REMARK** reporting guidelines (see link list at top right). See author guidelines, under 'Reporting Guidelines'. Please confirm you have followed these guidelines.	Not Applicable	
For **phase II and III randomized controlled trials**, please refer to the **CONSORT** flow diagram (see link list at top right) and submit the CONSORT checklist (see link list at top right) with your submission. See author guidelines, under 'Reporting Guidelines'. Please confirm you have submitted this list.	Not Applicable	

Data Availability

Data availability	Information included in the manuscript?	In which section is the information available? (Reagents and Tools Table, Materials and Methods, Figures, Data Availability Section)
Have **primary datasets** been deposited according to the journal's guidelines (see 'Data Deposition' section) and the respective accession numbers provided in the Data Availability Section?	Yes	Data Availability Section
Were **human clinical and genomic datasets** deposited in a public access-controlled repository in accordance to ethical obligations to the patients and to the applicable consent agreement?	Not Applicable	
Are **computational models** that are central and integral to a study available without restrictions in a machine-readable form? Were the relevant accession numbers or links provided?	Not Applicable	
If publicly available data were reused, provide the respective **data citations in the reference list**.	Yes	References, Appendix
